# Dissection of Ire1 Functions Reveals Stress Response Mechanisms Uniquely Evolved in *Candida glabrata*

**DOI:** 10.1371/journal.ppat.1003160

**Published:** 2013-01-31

**Authors:** Taiga Miyazaki, Hironobu Nakayama, Yohsuke Nagayoshi, Hiroshi Kakeya, Shigeru Kohno

**Affiliations:** 1 Department of Molecular Microbiology and Immunology, Nagasaki University School of Medicine, Nagasaki, Japan; 2 Faculty of Pharmaceutical Sciences, Suzuka University of Medical Sciences, Mie, Japan; David Geffen School of Medicine at University of California Los Angeles, United States of America

## Abstract

Proper protein folding in the endoplasmic reticulum (ER) is vital in all eukaryotes. When misfolded proteins accumulate in the ER lumen, the transmembrane kinase/endoribonuclease Ire1 initiates splicing of *HAC1* mRNA to generate the bZIP transcription factor Hac1, which subsequently activates its target genes to increase the protein-folding capacity of the ER. This cellular machinery, called the unfolded protein response (UPR), is believed to be an evolutionarily conserved mechanism in eukaryotes. In this study, we comprehensively characterized mutant phenotypes of *IRE1* and other related genes in the human fungal pathogen *Candida glabrata*. Unexpectedly, Ire1 was required for the ER stress response independently of Hac1 in this fungus. *C. glabrata* Ire1 did not cleave mRNAs encoding Hac1 and other bZIP transcription factors identified in the *C. glabrata* genome. Microarray analysis revealed that the transcriptional response to ER stress is not mediated by Ire1, but instead is dependent largely on calcineurin signaling and partially on the Slt2 MAPK pathway. The loss of Ire1 alone did not confer increased antifungal susceptibility in *C. glabrata* contrary to UPR-defective mutants in other fungi. Taken together, our results suggest that the canonical Ire1-Hac1 UPR is not conserved in *C. glabrata*. It is known in metazoans that active Ire1 nonspecifically cleaves and degrades a subset of ER-localized mRNAs to reduce the ER load. Intriguingly, this cellular response could occur in an Ire1 nuclease-dependent fashion in *C. glabrata*. We also uncovered the attenuated virulence of the *C. glabrata* Δ*ire1* mutant in a mouse model of disseminated candidiasis. This study has unveiled the unique evolution of ER stress response mechanisms in *C. glabrata*.

## Author Summary

The majority of secretory and transmembrane proteins are structurally matured in the endoplasmic reticulum (ER). The accumulation of misfolded proteins in the ER (ER stress) activates the ER-resident stress transducer Ire1, which has two distinct outputs: the unfolded protein response (UPR) and regulated Ire1-dependent decay (RIDD). The UPR is a transcriptional response to increase the protein folding capacity of the ER. RIDD induces degradation of ER-localized mRNAs to reduce the ER load. To date, the UPR has been believed to be an evolutionarily conserved pathway in almost all eukaryotic species, while RIDD has been found only in metazoans. Recent studies in several pathogenic fungi revealed that the UPR is implicated in antifungal resistance and virulence, and thus it has attracted attention as a therapeutic target. Here, we demonstrate that the important fungal pathogen *Candida glabrata* has lost the canonical UPR, but instead possesses the RIDD pathway and is relatively tolerant to ER stress. The transcriptional response to ER stress was dependent mainly on calcium signaling mediated by the protein phosphatase calcineurin in *C. glabrata*. Our results provide novel insights into ER quality control mechanisms and are useful for understanding evolutionary biology and the development of antifungal agents targeting the UPR.

## Introduction

In eukaryotic cells, the majority of secretory and transmembrane proteins are folded and modified in the lumen of the endoplasmic reticulum (ER). Contingent on proper folding, they are either transported to the Golgi apparatus or degraded [1]. Impairment of these vital cellular machines can be caused by various factors, such as chemical compounds and mutations in genes involved in ER quality control, resulting in the accumulation of unfolded or misfolded proteins in the ER, collectively called ER stress [2], [3]. Unsolved ER stress has detrimental consequences for eukaryotic cells. For example, in humans, ER stress is implicated in the pathology of various diseases including metabolic disease, inflammation, neurodegenerative disorders, and cancer [4], [5]. It has also been revealed in several pathogenic fungi that ER quality control is important for antifungal resistance and virulence [6], [7], [8], [9], [10], [11].

In response to ER stress, eukaryotic cells activate a signaling pathway, termed the unfolded protein response (UPR), to induce a comprehensive gene expression program that adjusts the protein-folding capacity of the ER (reviewed in [2], [12]). In *Saccharomyces cerevisiae*, the ER-resident transmembrane stress transducer Ire1, which contains an ER luminal sensing domain and cytosolic protein kinase/endoribonuclease domains, is responsible for detecting unfolded proteins and triggering the UPR [13], [14]. Ire1 is activated by direct interaction with the unfolded proteins, resulting in the oligomerization of its luminal domain. Signaling is transduced to the cytosolic domain through the sequential steps of autophosphorylation and oligomerization of the endoribonuclease domain, leading to the activation of the Ire1 ribonucrease [15], [16]. Active Ire1 cleaves the *HAC1* mRNA to excise the intron, allowing translation of the basic-leucine zipper (bZIP) transcription factor Hac1 that subsequently induces transcription of the UPR target genes [17], [18]. ER-stressed cells attempt to reduce the load of abnormally folded proteins in the ER by facilitating protein folding (e.g. upregulation of genes encoding ER-resident chaperones and protein-modifying enzymes) and by translocating misfolded proteins from the ER to the cytosol where they are degraded by the proteasome. The latter mechanism is called ER-associated protein degradation (ERAD) (reviewed in [3]). An alternative mechanism of degradative response is autophagy, which degrades organelles including damaged ER. In addition to ER-resident chaperones and protein-modifying enzymes, many of the components that mediate ERAD and autophagy have also been identified as UPR targets [19], [20], [21].

In pathogenic fungi, the molecular basis of ER quality control has been poorly understood, but several recent studies in *Aspergillus fumigatus* and *Cryptococcus neoformans* have found that Ire1 and Hac1 homologs are key components of the UPR and are indeed required for the ER stress response [7], [8], [10]. Interestingly, these studies have also discovered that the UPR is implicated in fungal pathogenicity and antifungal resistance. In *A. fumigatus*, the loss of HacA/Hac1 results in increased susceptibility to caspofungin, amphotericin B, and azole antifungals, and decreased virulence in mouse models of invasive aspergillosis [10]. Recently, the necessity of IreA/Ire1 for *A. fumigatus* virulence has also been reported [8]. *C. neoformans* mutant strains lacking Ire1 or its downstream transcription factor Hxl1 display increased azole susceptibility, failure to grow at 37°C, and avirulence in a mouse model of systemic cryptococcosis [7]. It is also known in *Candida albicans* that Hac1 plays a role in hyphal development [11], and a Δ*ire1* mutant is hypersusceptible to caspofungin [6]. These observations shed light on the UPR as an attractive target for the development of novel antifungal therapies.

*Candida glabrata* has emerged as an important fungal pathogen due in part to its intrinsic or rapidly acquired resistance to azole antifungals such as fluconazole [22], [23]. In addition, recent surveillance data have revealed an increase of *C. glabrata* clinical isolates that display resistance to not only azoles, but also echinocandin-class antifungals [24], [25]. Considering the limitations of the currently available antifungals in clinical settings, there is an urgent need to develop an effective antifungal strategy for a broad range of fungal pathogens, including *C. glabrata*.

Since how *C. glabrata* cells deal with ER stress has not been explored, we first functionally characterized the *C. glabrata* Ire1 and Hac1 orthologs. It has been believed that the UPR mediated by the Ire1-Hac1 linear pathway is evolutionarily conserved in most eukaryotic species, but surprisingly, we found that *C. glabrata* Ire1 plays a role in the ER stress response in a Hac1-independent manner, despite the presence of an apparent *HAC1* ortholog. The present study revealed that *C. glabrata* has lost the canonical Ire1-Hac1 UPR, but has developed alternative mechanisms for ER quality control. In addition, our comprehensive analyses of Δ*ire1* mutant phenotypes revealed significant diversities of Ire1-mediated stress response mechanisms between *C. glabrata* and other fungi. Here, we describe the unique evolution of ER quality control systems in *C. glabrata*.

## Results

### Comparison of growth ability in the presence of ER stress between *S. cerevisiae*, *C. neoformans*, and *Candida* spp

The ability of fungal cells to cope with ER stress was assessed by monitoring cell growth in the presence and absence of two well-known ER stress inducers that interfere with protein folding in the ER by different mechanisms: tunicamycin (TM), an N-linked glycosylation inhibitor, and dithiothreitol (DTT), an inhibitor of disulfide bond formation. Compared to *S. cerevisiae* and *C. neoformans* wild-type strains, *C. glabrata* was relatively tolerant to both TM and DTT independently of its strain backgrounds and culture media, although *S. cerevisiae* displayed strain dependent susceptibilities (Figure S1). Other representative *Candida* species also exhibited higher tolerance to these agents than *S. cerevisiae* and *C. neoformans* with intriguing exceptions: *Candida krusei* was highly susceptible to TM, but not to DTT, while *Candida tropicalis* was hypersusceptible to DTT, but not to TM. These results imply that diverse aspects of ER stress response mechanisms may exist even in closely related yeast species. In this report, the following studies focused on *C. glabrata*, which is phylogenetically close to *S. cerevisiae*, but has gained increased tolerance to ER stress and pathogenic potential to humans.

### Identification and structural characterization of the *IRE1* and *HAC1* orthologs in *C. glabrata*

**Figure 1:**
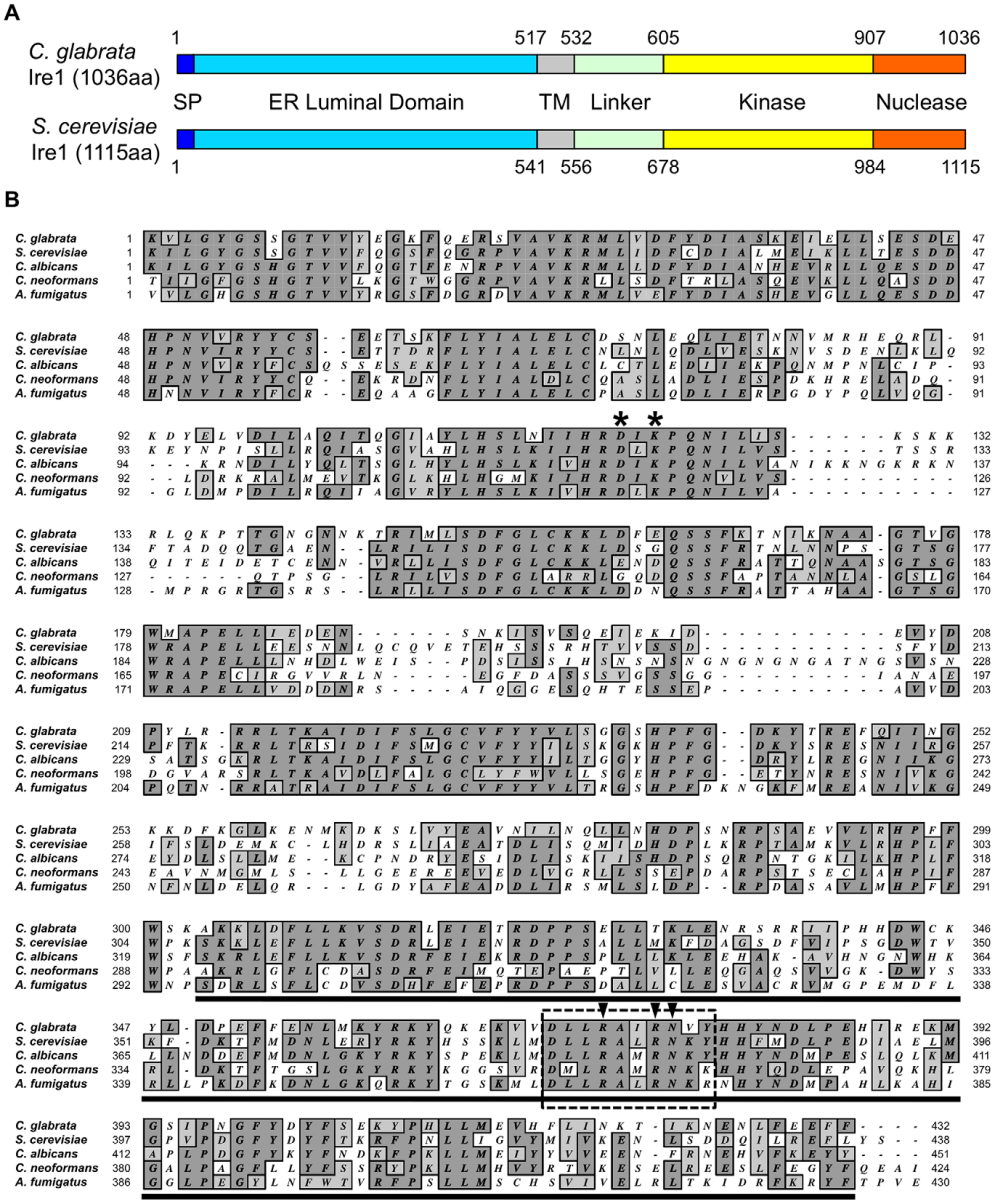
Sequence analysis of *C. glabrata* Ire1. (A) Schematic representation of the conserved domain structure of *C. glabrata* and *S. cerevisiae* Ire1. Abbreviations: SP, signal peptide; and TM, transmembrane. (B) Sequence alignment of the kinase and nuclease domains of fungal Ire1 orthologs. The asterisk indicates the conserved two catalytic residues in the nucleotide-binding pocket of Ire1 kinase (D797 and K799 in *S. cerevisiae* and D723 and K725 in *C. glabrata*). The predicted nuclease domain is underlined. Ten residues (boxed with dotted line) including the highly conserved three-nuclease active sites (arrowheads) are deleted in *C. glabrata IRE1*-ND. GenBank accession number: *Candida glabrata* Ire1, XP_446111; *Saccharomyces cerevisiae* Ire1, NP_011946; *Candida albicans* Ire1, XP_717532; *Cryptococcus neoformans* Ire1, XP_568837; and *Aspergillus fumigatus* IreA, AEQ59230.

The *C. glabrata IRE1* and *HAC1* orthologs were identified by BLASTp searches using the NCBI (http://www.ncbi.nlm.nih.gov/BLAST/) and Genolevures (http://cbi.labri.fr/Genolevures/elt/CAGL) databases. The respective amino acid sequences of *S. cerevisiae IRE1* (YHR079c) and *HAC1* (YFL031w) were used as queries. The deduced amino acid sequence of *C. glabrata IRE1* (NCBI accession No.: XP_446111, Genolevures ID: CAGL0F03245g) displayed 53.6% similarity and 35.8% identity with that of *S. cerevisiae IRE1*. As presented schematically in Figure 1A, *C. glabrata* Ire1 consists of typical Ire1-domain structures [16], [26], including an N-terminal hydrophobic signal sequence, an ER luminal domain, a transmembrane domain, and a serine-threonine protein kinase followed by a nuclease domain. The signal sequence and the ER luminal domain are important for ER localization and unfolded protein sensing, respectively [14], [27], [28], [29]. The most conserved segment is the cytoplasmic C-terminal region containing the protein kinase linked to the nuclease domain (Figure 1B). The protein kinase domain is required for autophosphorylation and concomitant activation of the nuclease domain, which provides the endoribonuclease activity [30], [31], [32]. *C. glabrata IRE1* is syntenic with *S. cerevisiae IRE1* situated on chromosome VIII, but the 3′ region of *C. glabrata IRE1* is orthologous to *S. cerevisiae* chromosome IV (Figure S2). This suggests that a genomic arrangement between *S. cerevisiae* chromosomes IV and VIII has occurred to evolve the *IRE1*-surrounding region on chromosome F in *C. glabrata*.

**Figure 2:**
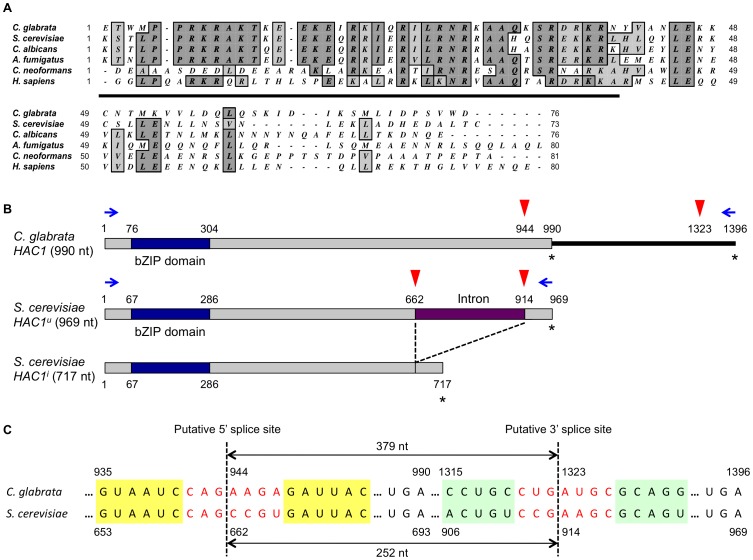
Sequence analysis of *C. glabrata* Hac1. (A) Sequence alignment of bZIP domains in Hac1 homologs from fungi and humans. The DNA binding domain is underlined. GenBank accession number: *Candida glabrata* Hac1, XP_448761; *Saccharomyces cerevisiae* Hac1, NP_116622; *Candida albicans* Hac1, XP_718538; *Aspergillus fumigatus* HacA, XP_748727; *Cryptococcus neoformans* Hxl1, XP_568439; and *Homo sapiens* Xbp1, NP_005071. (B) A schematic representation of the putative splice sites and ORF lengths in *C. glabrata* and *S. cerevisiae HAC1*. The *HAC1* ORFs and bZIP domains were shown in grey and blue boxes, respectively. In *S. cerevisiae*, the intron (purple box) in the uninduced form of *HAC1* (*HAC1^u^*) is excised in the induced form of *HAC1* (*HAC1^i^*). Red arrowheads and asterisks indicate putative splice sites and stop codons, respectively. Blue arrows indicate primers that were used for RT-PCR assays to examine *HAC1* splicing. (C) Alignment of the RNA sequence surrounding the predicted intron in *C. glabrata* and *S. cerevisiae HAC1*. In *S. cerevisiae*, *HAC1* mRNA is known to form stem-loop structures that enclose the intron between the two loops of seven residues (shown in red) held in place by short stems (yellow and green boxes). The predicted 5′ and 3′ splice sites and intron lengths in the *S. cerevisiae* and *C. glabrata HAC1* mRNAs are indicated.

A BLASTp search using the NCBI and Genolevures databases also identified a single *HAC1* ortholog (NCBI accession No.: XP_448761, Genolevures ID: CAGL0K12540g) in the *C. glabrata* genome, exhibiting 36.9% similarity and 24.2% identity with the deduced amino acid sequence of the unspliced form of *S. cerevisiae HAC1*. It has been known that functional orthologs of transcription factors often display limited sequence similarity outside their DNA-binding domains [11]. Indeed, *C. glabrata HAC1* is not highly conserved at the overall sequence level, but contains a bZIP domain at the N-terminal region. The putative DNA binding domain of *C. glabrata* Hac1 displays strong sequence similarity to those of other Hac1 homologs (Figure 2A). In addition, there is a clear syntenic relationship between *S. cerevisiae* and *C. glabrata HAC1* loci (Figure S2). Based on these sequence similarities and synteny conservation, we considered the genes CAGL0F03245g and CAGL0K12540g to be *C. glabrata IRE1* and *HAC1*, respectively.

It has been known in *S. cerevisiae* [33], [34], [35] that the uninduced form of *HAC1* (*HAC1^u^*) contains the 252 nucleotides intron, which is excised by Ire1 to generate the induced form of *HAC1* (*HAC1^i^*) (Figure 2B). The well-conserved Ire1 recognition motifs, CNG′CNGN and CNG′AAGC at the 5′ and 3′ boundaries of the intron, respectively [36], locate in the loop regions of the *HAC1* mRNA stem-loop structures [34], [37]. Based on the conservation of common features around the predicted intron (Figure 2C), *C. glabrata HAC1* mRNA is also likely to form stem-loop structures [36]. If excision of the predicted intron in *C. glabrata HAC1* mRNA occurs, it changes the last 15 amino acids of the putative ORF and extends it by only 9 amino acids (Figures 2B and 2C). However, *C. glabrata HAC1* has mutations in the consensus sequences of the Ire1 recognition motifs at both splice sites: a C to A mutation at the 4th position of the 5′ splice site and an A to U mutation at the 5th position of the 3′ splice site (Figure 2C).

### Ire1, but not Hac1, is required for ER stress response in *C. glabrata*

**Figure 3:**
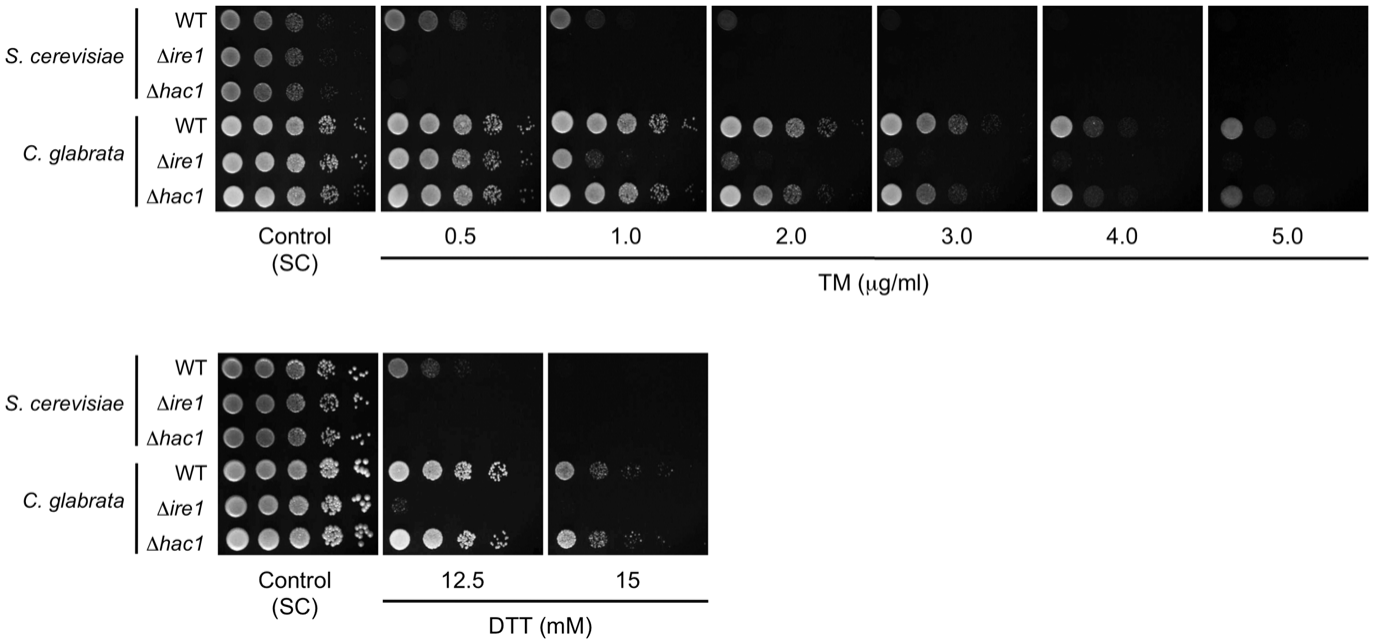
Growth assay in the presence of ER stress-inducing agents. Logarithmic-phase cells of *S. cerevisiae* and *C. glabrata* strains were adjusted to 2×10^7^ cells/ml, and then 5 µl of serial 10-fold dilutions were spotted onto synthetic complete (SC) plates containing either tunicamycin (TM) or dithiothreitol (DTT) at the indicated concentrations. Plates containing TM and DTT were incubated at 30°C for 24 and 48 h, respectively. *S. cerevisiae* strains: WT, BY4742; Δ*ire1*, BY4742Δire1; and Δ*hac1*, BY4742Δhac1. *C. glabrata* strains: WT, CBS138; Δ*ire1*, TG121; and Δ*hac1*, TG141.

To examine involvement of Ire1 and Hac1 in the ER stress response in *C. glabrata*, we constructed a mutant of each in which the entire open reading frame (ORF) of *IRE1* or *HAC1* was deleted, and examined growth on plates containing TM and DTT (Figure 3). Both *S. cerevisiae* Δ*ire1* and Δ*hac1* mutants were used as controls and they exhibited severe growth defects in the presence of TM or DTT. In *C. glabrata*, the Δ*ire1* mutant displayed decreased tolerance to both TM and DTT compared with the wild-type strain, whereas surprisingly, the Δ*hac1* mutant exhibited wild-type growth in the presence of these ER stress-inducing agents (Figure 3). Southern blot analysis demonstrated that *HAC1* was truly absent and there was no ectopic integration of the deletion construct in the Δ*hac1* mutant (Figure S3). The results indicate that Ire1, but not Hac1, is required for the ER stress response in *C. glabrata*. This is quite a contrast to *S. cerevisiae*, which is dependent equally on Ire1 and Hac1 to survive ER stress [38], implying that *C. glabrata* may possess a unique mechanism, which is mediated by Ire1 independently of Hac1, to cope with ER stress.

### Ire1 does not cleave *HAC1* mRNA in *C. glabrata*

**Figure 4:**
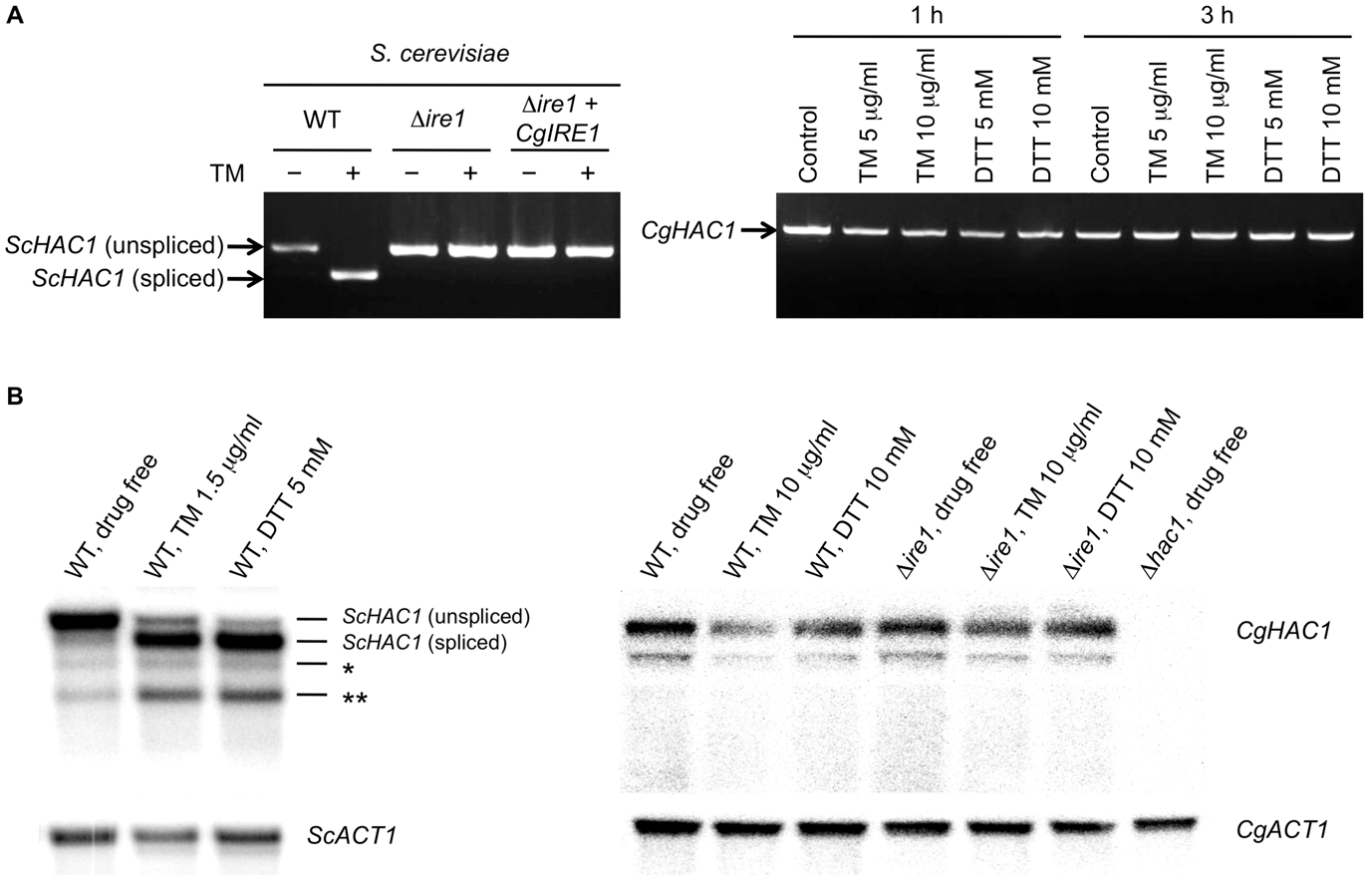
Assays for *HAC1* mRNA splicing. (A) RT-PCR analysis. The *S. cerevisiae* strains containing either empty vector or pRS415-ADH-CgIRE1, in which *C. glabrata IRE1* was expressed under the control of the *S. cerevisiae ADH1* promoter, were incubated in SC-leu broth in the presence and absence of 1.5 µg/ml tunicamycin (TM) for 3 h. *S. cerevisiae* strains: WT, BY42-1; Δ*ire1*, BY42I-1; and Δ*ire1*+*CgIRE1*, BY42I-2. The *C. glabrata* wild-type strain CBS138 was incubated in SC broth in the presence and absence of TM and dithiothreitol (DTT) at the indicated concentrations for 1 and 3 h. RT-PCR products of the entire *HAC1* mRNA in *S. cerevisiae* (left panel) and *C. glabrata* (right panel) were electrophoresed on a 1% agarose gel. (B) Northern blot analysis. *S. cerevisiae* WT (BY4742) cells were treated with 1.5 µg/ml TM or 5 mM DTT for 1 h (left panel). *C. glabrata* cells were treated with 10 µg/ml TM or 10 mM DTT for 3 h (right panel). Both *ScHAC1* and *CgHAC1* probes were generated from the 5′ regions of the *HAC1* ORFs. The same blot was probed for *HAC1* mRNA, stripped, and then probed for *ACT1* mRNA. The asterisks indicate potential splicing intermediates (*: 5′ exon plus the intron; **: 5′ exon alone). *C. glabrata* strains: WT, CBS138; Δ*ire1*, TG121; and Δ*hac1*, TG141.

Ire1-mediated splicing of *HAC1*/*XBP1* mRNA was first uncovered in *S. cerevisiae* [32], and similar mechanisms in *HAC1*/*XBP1* homologs were later described in some fungi, including *Trichoderma reesei*, *Aspergillus nidulans* [39], *C. albicans* [11], *Pichia pastoris* [40], *Yarrowia lipolytica* [41], *A. fumigatus* [8], [10], and *C. neoformans* [7], as well as in metazoans including *Caenorhabditis elegans*, mice [37], [42], humans [43], and *Drosophila melanogaster* [44]. We examined *HAC1* mRNA splicing in ER-stressed *S. cerevisiae* and *C. glabrata* cells by RT-PCR using the *HAC1* specific primer pairs denoted in Figure 2B. As expected, *S. cerevisiae HAC1* mRNA was spliced in response to treatment with 1.5 µg/ml TM in wild-type control but not in the Δ*ire1* strain (Figure 4A, left panel). As *C. glabrata* wild-type cells were relatively tolerant to both TM and DTT (Figure S1), they were treated with higher concentrations of TM and DTT for 1 and 3 h. However, contrary to *S. cerevisiae*, splicing of *HAC1* mRNA was not induced in *C. glabrata* under any of the conditions tested in this study (Figure 4A, right panel). The absence of a *HAC1* splicing event was also verified by sequencing the RT-PCR products of the entire *HAC1* mRNA (data not shown), suggesting that Hac1 is not a downstream target of Ire1 in *C. glabrata*.

To further confirm this aspect, we also performed Northern blot analysis using the probes directed against the 5′ region of the *HAC1* ORF in *S. cerevisiae* and *C. glabrata*. In *S. cerevisiae*, *HAC1* mRNA was efficiently spliced upon ER stress induced by 1.5 µg/ml TM or 5 mM DTT (Figure 4B, left panel). In addition to the unspliced and spliced *HAC1* mRNAs, detection of two smaller bands (indicated with asterisks in Figure 4B) were consistent with previous reports [17], [45], [46], although it is unclear whether these bands correspond to splicing intermediates or dead-end products [17], [46]. The *C. glabrata* wild-type and Δ*ire1* cells were incubated under the normal conditions and in the presence of 10 µg/ml TM or 10 µM DTT for 3 h. Under all conditions tested, the *C. glabrata* wild-type strain somehow exhibited 2 bands but these bands were also detected similarly in the Δ*ire1* mutant (Figure 4B, right panel), indicating that it was not due to Ire1-mediated splicing. These results and the phenotypic differences between the Δ*ire1* and Δ*hac1* mutants strongly suggest that *C. glabrata* Ire1 plays a role in the ER stress response in a Hac1-independent manner.

### Exploration of other bZIP transcription factors as potential downstream targets of Ire1 in *C. glabrata*

To address the possibility that a *HAC1*-like gene encoding a bZIP transcription factor may exist as a downstream target of Ire1 in *C. glabrata*, we performed a low-stringent BLASTp search (e-value = 10^−2^) using only the bZIP domain sequences of *S. cerevisiae* Hac1, *C. neoformans* Hxl1, and *Homo sapiens* Xbp1 as described previously [7]. In addition to *HAC1*, the following eight putative genes were identified in the *C. glabrata* genome (Table S1): CAGL0H04631g (*YAP1*), CAGL0J06182g (*SKO1*), CAGL0K02585g (*YAP3*), CAGL0M10087g (*YAP3*), CAGL0L02475g (*GCN4*), CAGL0F03069g (*CAD1*/*YAP2*), CAGL0F01265g (*YAP7*), and CAGL0M08800g (*YAP6*). We sequenced the RT-PCR products of these mRNAs, which were obtained from *C. glabrata* wild-type cells treated with TM at concentrations of 1.5 and 5 µg/ml for 3 h, but none of them displayed evidence of a splicing event (data not shown). The results suggest that another Hac1 homolog is unlikely to exist in *C. glabrata*.

### Complementation assays of the *IRE1* and *HAC1* orthologs between *S. cerevisiae* and *C. glabrata*

**Figure 5:**
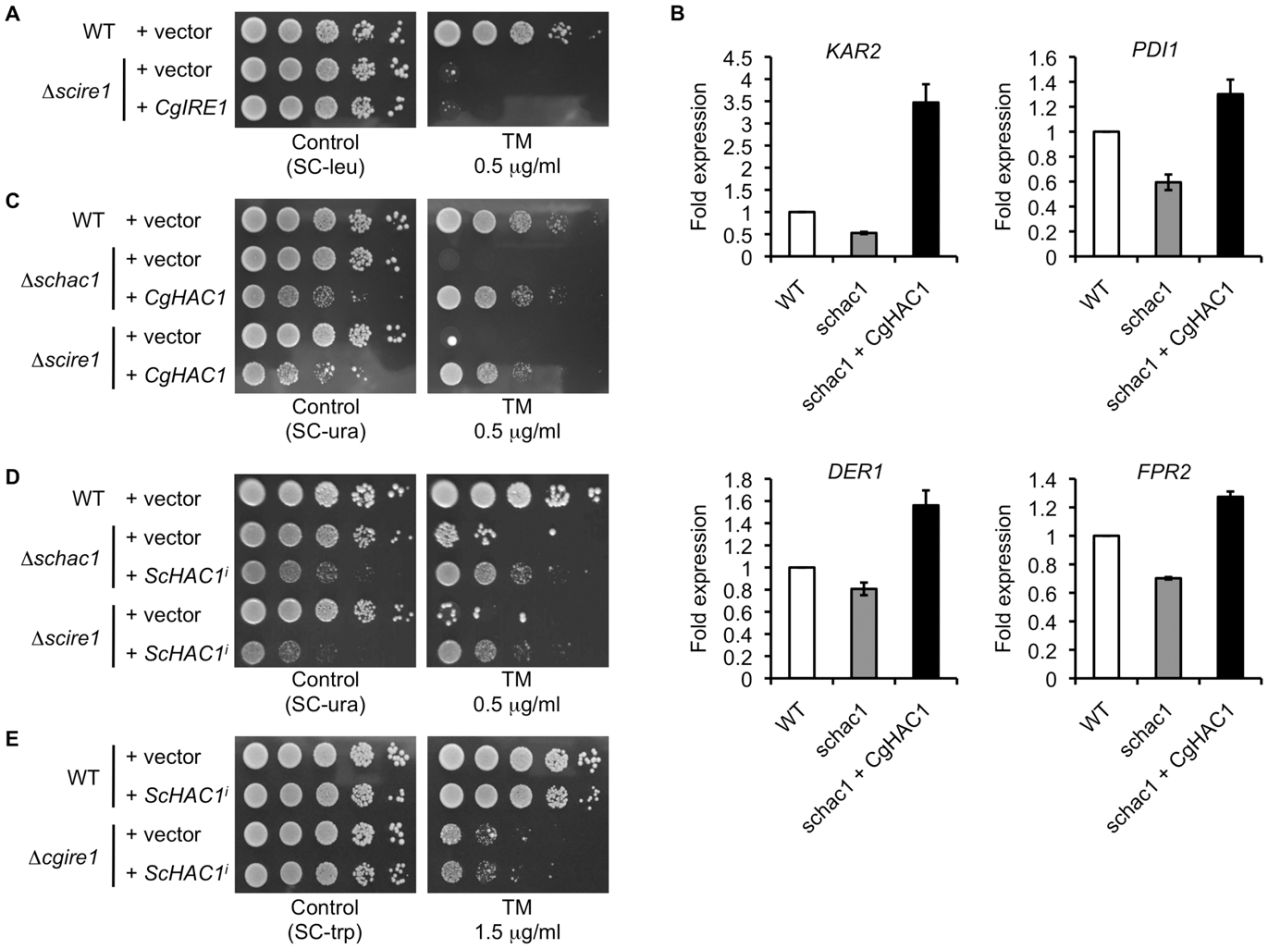
Functional complementation assays of the *IRE1* and *HAC1* orthologs in *S. cerevisiae* and *C. glabrata*. (A) *C. glabrata IRE1* was expressed under the control of the *S. cerevisiae ADH1* promoter in the *S. cerevisiae* Δ*ire1* mutant. Logarithmic-phase cells were adjusted to 2×10^7^ cells/ml, and then 5 µl of serial 10-fold dilutions were spotted onto agar plates in the presence and absence of 0.5 µg/ml tunicamycin (TM). Plates were incubated at 30°C for 48 h. *S. cerevisiae* strains: WT+vector, BY42-1; Δ*scire1*+vector, BY42I-1; and Δ*scire1*+*CgIRE1*, BY42I-2. (B) *C. glabrata HAC1* was expressed under the control of the *S. cerevisiae ADH1* promoter in the *S. cerevisiae* Δ*hac1* mutant. Expression levels of representative UPR target genes in *S. cerevisiae* were analyzed by qRT-PCR as described in Materials and Methods. Results are presented as fold expression relative to the levels in the wild-type control. The means and standard deviations for three independent experiments are shown. *S. cerevisiae* strains: WT, BY42-2; schac1, BY42H-1; and schac1+CgHAC1, BY42H-2. (C) *C. glabrata HAC1* was expressed under the control of the *S. cerevisiae ADH1* promoter in the *S. cerevisiae* Δ*hac1* and Δ*ire1* mutants. The assay was performed as in part A. *S. cerevisiae* strains: WT+vector, BY42-2; Δ*schac1*+vector, BY42H-1; and Δ*schac1*+*CgHAC1*, BY42H-2; Δ*scire1*+vector, BY42I-3; and Δ*scire1*+*CgHAC1*, BY42I-4. (D) The induced form of *S. cerevisiae HAC1*, denoted as *ScHAC1^i^*, was expressed under the control of the *S. cerevisiae PGK1* promoter in the *S. cerevisiae* Δ*hac1* and Δ*ire1* mutants. The assay was performed as in part A. *S. cerevisiae* strains: WT+vector, BY42-2; Δ*schac1*+vector, BY42H-1; and Δ*schac1*+*ScHAC1^i^*, BY42H-3; Δ*scire1*+vector, BY42I-3; and Δ*scire1*+*ScHAC1^i^*, BY42I-5. (E) *ScHAC1^i^* was expressed under the control of the *S. cerevisiae PGK1* promoter in the *C. glabrata* wild-type and Δ*ire1* mutant strains. The assay was performed as in part A except TM concentration (1.5 µg/ml). *C. glabrata* strains: WT+vector, TG11; WT+*ScHAC1^i^*, TG13; Δ*cgire1*+vector, TG122; and Δ*cgire1*+*ScHAC1^i^*, TG126.

To investigate whether *C. glabrata* Ire1 could complement *S. cerevisiae* Ire1 function, *C. glabrata IRE1* was expressed under the *S. cerevisiae ADH1* promoter in the *S. cerevisiae* Δ*ire1* mutant. After treatment with 1.5 µg/ml TM for 3 h, a spliced form of *S. cerevisiae HAC1* was generated in the wild-type strain, but not in the Δ*ire1* strain regardless of the presence of *C. glabrata IRE1* (Figure 4A). The absence of a *HAC1* splicing event in the *S. cerevisiae* Δ*ire1* mutant containing *C. glabrata IRE1* was also verified by sequencing analyses of the RT-PCR products (data not shown). As expected, *C. glabrata* Ire1 did not rescue the growth of the *S. cerevisiae* Δ*ire1* mutant in the presence of TM (Figure 5A). These results indicate that *C. glabrata* Ire1 is unable to initiate the UPR due to its inability to cleave the *HAC1* mRNA in *S. cerevisiae*, accounting for the growth defects of the *S. cerevisiae* Δ*ire1* mutant containing *C. glabrata IRE1* in the presence of TM.

Although *HAC1* was dispensable for *C. glabrata* cells to cope with ER stress (Figure 3), the fact that the bZIP domain is conserved in this gene prompted us to investigate whether *C. glabrata* Hac1 behaves as a transcription factor similarly to *S. cerevisiae* Hac1. To address this question, *C. glabrata HAC1* was constitutively expressed under the *ADH1* promoter in the *S. cerevisiae* Δ*hac1* mutants, and the transcription of representative UPR target genes, including *KAR2* (ER-resident chaperone), *PDI1* (protein disulfide isomerase), *DER1* (ER-associated degradation), and *FPR2* (ER protein trafficking), was examined. Compared to the wild-type strain, the expression levels of these genes were decreased in the Δ*hac1* mutant, but reversed by the heterologous expression of *C. glabrata HAC1* in the mutant (Figure 5B). Although a direct interaction between *C. glabrata* Hac1 and UPR targets in *S. cerevisiae* was not examined, the overexpression of *C. glabrata HAC1* conferred the increased expression levels of these genes (e.g. 3.5-fold induction of *KAR2* expression relative to the wild-type control). Since sustained activation of the UPR is toxic to cells [47], the constitutive overexpression of *C. glabrata HAC1* and the induced form of *S. cerevisiae HAC1* (*ScHAC1^i^*) similarly impaired cell growth even under normal growth conditions (Figures 5C and 5D). Furthermore, growth of the *S. cerevisiae* Δ*hac1* mutant in the presence of TM was rescued by *C. glabrata* Hac1 to the wild-type level (Figure 5C). The sequences of *C. glabrata HAC1* obtained by RT-PCR from TM-treated and -untreated cells were identical (data not shown). These results indicate that *C. glabrata* Hac1 was matured without splicing in *S. cerevisiae*. In agreement with this, *C. glabrata* Hac1 also rescued growth of the *S. cerevisiae* Δ*ire1* mutant in the presence of TM (Figure 5C). These results suggest that *C. glabrata* Hac1 retains a function as a transcription factor to activate the UPR targets; however, unlike *S. cerevisiae HAC1*, the *C. glabrata HAC1* mRNA does not need to be spliced by Ire1 before translation.

We also examined the effects of heterologous expression of *S. cerevisiae HAC1* in *C. glabrata*. Since *C. glabrata* Ire1 was unable to splice *S. cerevisiae HAC1* (Figure 4A), *ScHAC1^i^* was expressed under the control of the *PGK1* promoter in *C. glabrata*. Although *C. glabrata* Hac1 complemented *S. cerevisiae* Hac1 functions (Figures 5B and 5C), *ScHAC1^i^* did not rescue growth of the *C. glabrata* Δ*ire1* mutant in the presence of TM (Figure 5E). *ScHAC1^i^* partially rescued growth of the *S. cerevisiae* Δ*hac1* and Δ*ire1* mutants in the presence of TM, confirming its functionality (Figure 5D). Since *C. glabrata* Hac1 induced transcription of the UPR targets in *S. cerevisiae* (Figure 5B), the Hac1 orthologs in *C. glabrata* and *S. cerevisiae* may share a common binding motif in the promoter regions of the target genes. These results support the idea that the impaired ER stress response of the *C. glabrata* Δ*ire1* mutant is not associated with Hac1 functions.

### The Ire1, calcineurin, and Slt2 signaling pathways are coordinately required for ER stress response in *C. glabrata*

**Figure 6:**
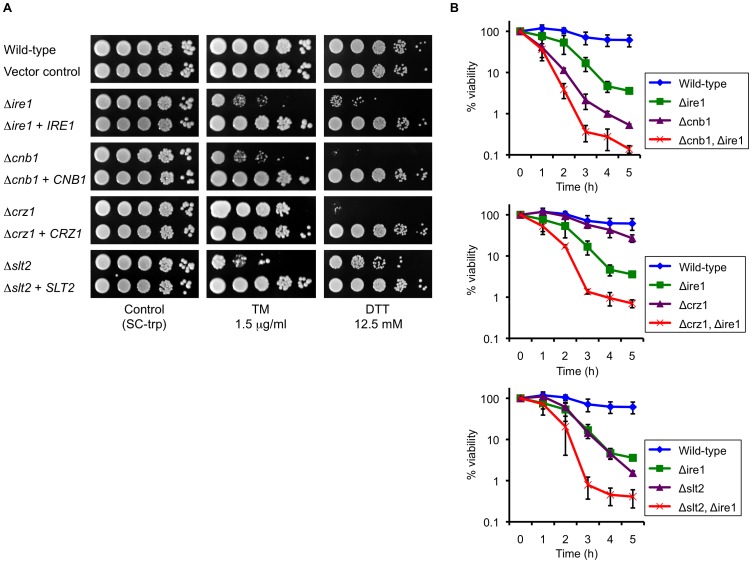
Multiple signaling pathways are coordinately involved in the ER stress response in *C. glabrata*. (A) The *C. glabrata* Δ*ire1*, Δ*cnb1*, Δ*crz1*, and Δ*slt2* deletion mutants were transformed with either an empty vector or a plasmid containing the corresponding wild-type gene. Logarithmic-phase cells were adjusted to 2×10^7^ cells/ml, and then 5 µl of serial 10-fold dilutions were spotted onto synthetic complete medium without tryptophan (SC-trp) plates in the presence and absence of tunicamycin (TM) and dithiothreitol (DTT) at the indicated concentrations. Plates were incubated at 30°C for 48 h. (B) Time-kill analysis of the *C. glabrata* deletion mutants in the presence of TM. Logarithmic-phase cells (5×10^5^ CFU/ml) were incubated in SC medium containing 1.5 µg/ml TM. The number of viable cells was determined by plating the appropriate dilutions on YPD plates at the indicated time points. Data are expressed as the percentages of viability relative to the untreated (time point 0) control in each strain. The means and standard deviations for three independent experiments are shown. *C. glabrata* strains: Wild-type, CBS138; Vector control, TG11; Δ*ire1*, TG122; Δ*ire1*+*IRE1*, TG123; Δ*cnb1*, TG162; Δ*cnb1*+*CNB1*, TG163; Δ*crz1*, TG172; Δ*crz1*+*CRZ1*, TG173; Δ*slt2*, TG152; Δ*slt2*+*SLT2*, TG153; Δ*cnb1* Δ*ire1*, TG1612; Δ*crz1* Δ*ire1*, TG1712; and Δ*slt2* Δ*ire1*, TG1512.

We next investigated the possibility that the cellular response to ER stress might involve potential crosstalk between Ire1 and other signaling pathways in *C. glabrata*. In addition to the UPR, the serine-threonine-specific protein phosphatase calcineurin and the Slt2 mitogen-activated protein kinase (MAPK) pathway are also required for the ER stress response in *S. cerevisiae* [48], [49], [50], [51]. We examined whether this is the case in *C. glabrata* using four *C. glabrata* mutant strains lacking a key component of these signaling pathways, including Ire1, the regulatory B subunit of calcineurin Cnb1, the calcineurin-regulated transcription factor Crz1, and the last member of the PKC1-MAPK cascade Slt2. While all mutants displayed wild-type growth on a drug-free control plate, the Δ*ire1*, Δ*cnb1*, and Δ*slt2* mutants exhibited decreased tolerance to both TM and DTT (Figure 6A). Growth of the Δ*crz1* mutant was at nearly wild-type levels in the presence of TM, but drastically impaired on the plate containing DTT. These results were in agreement with a previous proposal that *C. glabrata* calcineurin is involved in various stress responses via Crz1-dependent and -independent mechanisms depending on the type of stress [52]. All mutant phenotypes were recovered to wild-type levels by reintroducing the corresponding wild-type genes into the mutants (Figure 6A). Taken together, the results indicate that Ire1, calcineurin, and Slt2 are required for the ER stress response in *C. glabrata*, consistent with previous findings in *S. cerevisiae*.

Next, we deleted *IRE1* in the Δ*cnb1*, Δ*crz1*, and Δ*slt2* backgrounds and monitored the viabilities of the single and double deletants after exposure to 1.5 µg/ml TM in liquid medium for up to 5 h. The double deletants displayed more rapid and greater reduction of viability than each single deletant in the presence of TM (Figure 6B). The results suggest that Ire1, calcineurin, and Slt2 function in parallel to cope with ER stress in *C. glabrata*.

### Gene expression profiling of *C. glabrata* mutants in the presence of ER stress

**Figure 7:**
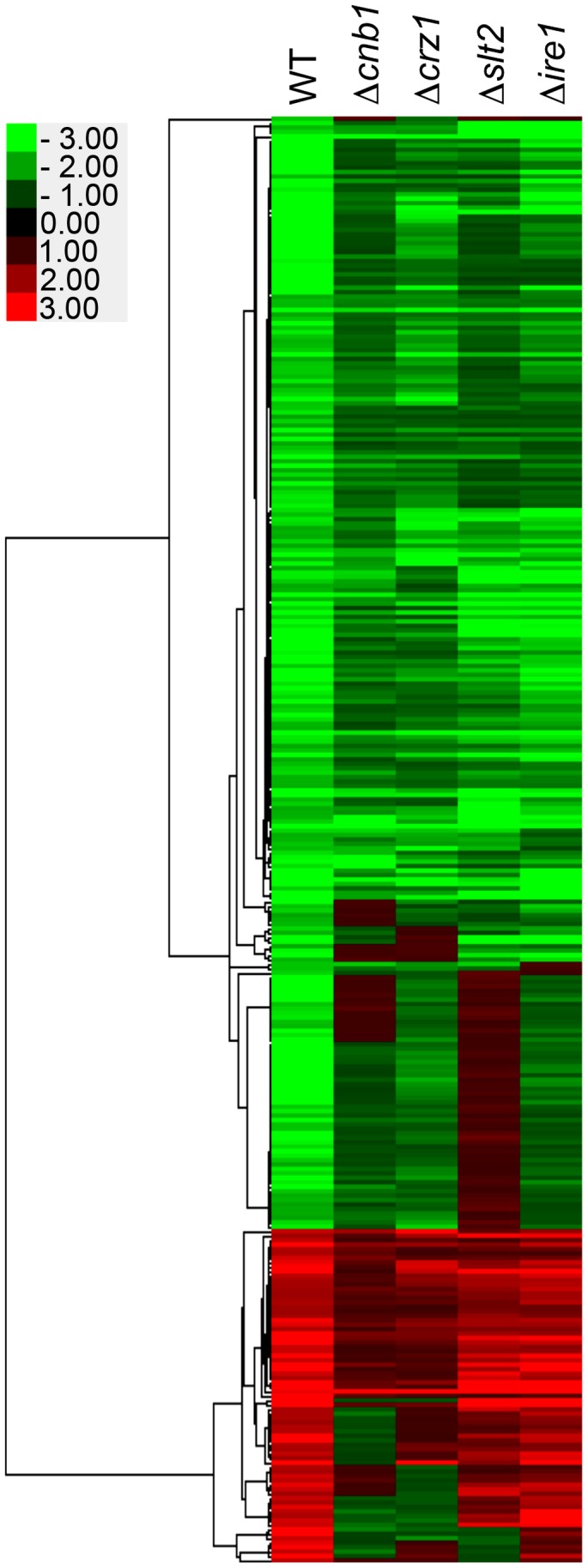
Genome-wide gene expression profiles in response to tunicamycin (TM) exposure for 3 h. Hierarchical clustering of genes whose expression levels were changed more than 2-fold after treatment with 1.5 µg/ml TM for 3 h. Genes were clustered with centroid linkage. *C. glabrata* strains: WT, 2001T; Δ*cnb1*, TG161; Δ*crz1*, TG171; Δ*slt2*, TG151; and Δ*ire1*, TG121.

To examine the transcriptional response to ER stress in *C. glabrata*, the wild-type, Δ*ire1*, Δ*cnb1*, Δ*crz1*, and Δ*slt2* mutants were exposed to 1.5 µg/ml TM for 0.5 and 3 h, and genome-wide analyses were conducted using DNA microarrays. The complete dataset can be found at the NCBI Gene Expression Omnibus (GEO, http://www.ncbi.nlm.nih.gov/geo/) with accession number GSE29855. It has been reported that at least 381 genes, corresponding to more than 5% of the ORFs in the genome, were induced in response to TM and DTT under the control of the UPR in *S. cerevisiae* [20]. In our assay, a total of 325 genes were differentially regulated (greater than 2.0-fold change) and 75 genes of them were upregulated in the *C. glabrata* wild-type strain after treatment with TM for 3 h (Figure 7 and Table S2). In contrast to previous findings in *S. cerevisiae* [20], the induced dataset in *C. glabrata* was not enriched for genes involved in folding capacity of the ER (Table S2). Therefore, to rule out the possibility that unfolded protein stress might not be induced sufficiently by the conditions used in our study, *C. glabrata* wild-type cells were treated with higher concentrations of TM and DTT, and then expression levels of five *C. glabrata* genes (*KAR2*, *PDI1*, *DER1*, *FPR2* and *HAC1*), which are well-known UPR targets in *S. cerevisiae* [20], [53], were examined by qRT-PCR. The induction levels of *KAR2* in the presence of high concentrations of TM and DTT were similar to those observed in the presence of 1.5 µg/ml TM (Figure S4A). In addition, expression levels of the other 4 genes were not increased even in cells treated with high concentrations of TM and DTT (Figure S4B). Collectively, these results suggest that ER quality may be controlled differently in *C. glabrata* than *S. cerevisiae*, which relies primarily on transcriptional induction of genes that increase the protein folding capacity of the ER.

**Figure 8:**
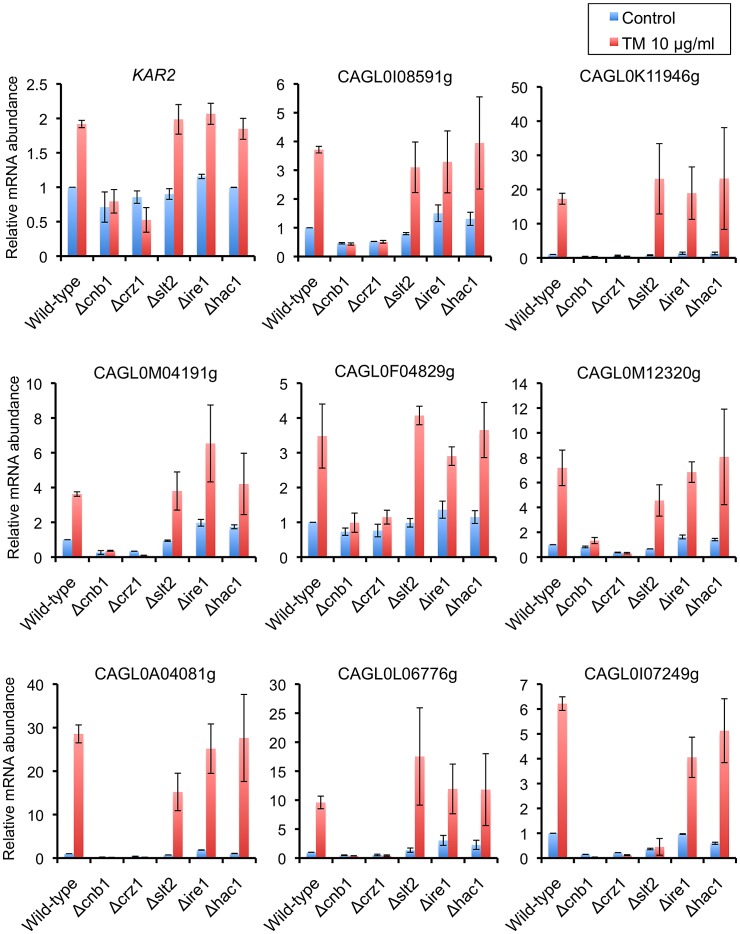
qRT-PCR validation of transcriptional profiles in the presence of tunicamycin (TM). Logarithmic-phase *C. glabrata* cells were incubated in the presence and absence of 10 µg/ml TM. qRT-PCR was performed as described in Materials and Methods. The means and standard deviations for three independent experiments are shown. *C. glabrata* strains: WT, 2001T; Δ*cnb1*, TG161; Δ*crz1*, TG171; Δ*slt2*, TG151; Δ*ire1*, TG121; and Δ*hac1*, TG141.

Furthermore, in contrast to *S. cerevisiae*, the vast majority of genes induced in the *C. glabrata* wild-type strain were also similarly upregulated in the Δ*ire1* mutant, but at less than two-fold change, or were downregulated in the Δ*cnb1* mutant (Figure 7 and Table S2). The loss of Crz1 and Slt2 had partial effects on the upregulation of those genes. Many of the genes induced by TM overlapped with calcineurin-regulated genes reported in a recent study of calcineurin signaling in *C. glabrata* [54], indicating the activation of calcineurin signaling in response to TM. A subset of genes that displayed calcineurin-dependent upregulation in the presence of 1.5 µg/ml TM in our microarray experiments were further evaluated by qRT-PCR after treatment with a higher concentration of TM (10 µg/ml) for 3 h (Figure 8). All the genes examined in this assay were upregulated upon TM treatment in a calcineurin- and Crz1-dependent manner but independently of Ire1 and Hac1. In addition to calcineurin, Slt2 was also required for the induction of CAGL0I07249g, consistent with the microarray data. Although these assays cannot differentiate a direct from an indirect effect, the results suggest that calcineurin, but not Ire1, plays important roles in transcriptional response to ER stress in *C. glabrata*.

Transcriptional induction of the ER-resident chaperone *KAR2* is a well-known marker for UPR activation in yeast [55], [56]. In response to TM treatment, expression levels of *C. glabrata KAR2* were increased in the wild-type, Δ*slt2*, and Δ*ire1* strains, but not in the Δ*cnb1* and Δ*crz1* mutants (Figure 8). These results are in contrast to previous observations in *S. cerevisiae*, where Ire1-Hac1 signaling is required for upregulation of *KAR2* in response to ER stress [13], [17]. A Hac1-binding site, termed the unfolded protein response element (UPRE), was originally defined as a 22-bp sequence element of the *S. cerevisiae KAR2* promoter and subsequently refined to a seven-nucleotide consensus, 5′-CAGNGTG-3′ [57], [58]. Mutations in any of the six conserved nucleotides, or deletion of the central nucleotide, cause it to lose its function as an autonomous upstream activating sequence [57]. Consistent with the *KAR2* expression data, the consensus UPRE sequence was absent in the 1-kb upstream region of *C. glabrata KAR2*. On the other hand, it is known in *S. cerevisiae* and *C. glabrata* that the promoters of most calcineurin-dependent genes contain one to six copies of the Crz1-binding sequence, 5′-GNGGC(G/T)-3′ [59], [60]. Five copies of the Crz1-binding sequence, including one copy of the full consensus sequence, 5′-GNGGCTCA-3′ [60], were found within the 1-kb upstream region of *C. glabrata KAR2*. These results support our *KAR2* expression data, collectively suggesting that transcriptional induction of *KAR2* in response to ER stress is mediated by the calcineurin-Crz1 pathway, but not by Ire1 signaling, in *C. glabrata*.

In our microarray analyses, there was a subset of ∼33 genes whose mRNA abundance was decreased in the wild-type strain, but not in the Δ*ire1* mutant at a relatively early phase (0.5 h) of ER stress (Figures S5 and Table S4). This cluster predominantly contained genes encoding membrane proteins involved in transferase activity (Table S5). Most of these gene products are known to be ER-resident or pass through the ER before translocating to their final destinations. Further analyses were made to interpret this phenomenon, which will be discussed below.

### Functional dissection of the protein kinase and nuclease domains in *C. glabrata* Ire1

**Figure 9:**
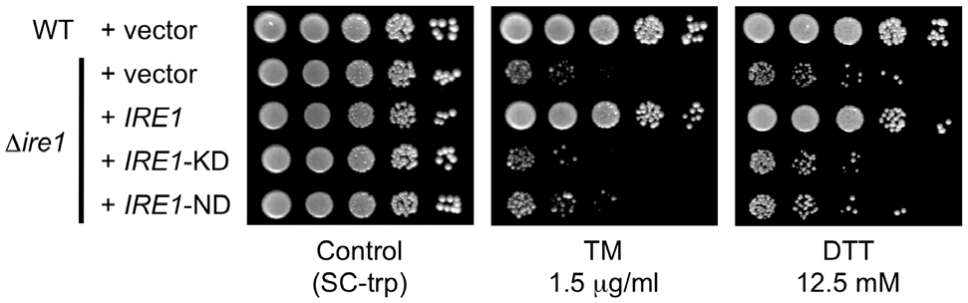
Dissection of Ire1 functions required for the ER stress response in *C. glabrata*. (A) Both the kinase and nuclease activity of Ire1 are required for cell growth in the presence of ER stress. The *C. glabrata* Δ*ire1* mutant was transformed with an empty vector or a plasmid containing wild-type *IRE1*, kinase-dead *IRE1* (*IRE1*-KD), or nuclease-dead *IRE1* (*IRE1*-ND). Logarithmic-phase cells were adjusted to 2×10^7^ cells/ml, and then 5 µl of serial 10-fold dilutions were spotted onto synthetic complete medium without tryptophan (SC-trp) plates in the presence and absence of tunicamycin (TM) and dithiothreitol (DTT) at the indicated concentrations. Plates were incubated at 30°C for 48 h. *C. glabrata* strains: WT+vector, TG11; Δ*ire1*+vector, TG122; Δ*ire1*+*IRE1*, TG123; Δ*ire1*+*IRE1*-ND, TG125; and Δ*ire1*+*IRE1*-KD, TG124.

To further investigate how Ire1 is involved in the ER stress response in *C. glabrata*, we examined the contribution of the protein kinase and nuclease functions of *C. glabrata* Ire1 to cell growth in the presence of TM and DTT. In *S. cerevisiae*, mutations of two catalytic residues (D797 and K799) in the nucleotide-binding pocket of Ire1 kinase to asparagines abolish phosphorylation, but preserve RNase activity [61]. Therefore, these mutations (D797N, K799N) allow uncoupling of kinase and nuclease activities of Ire1. Since these two residues are highly conserved in the Ire1 orthologs of fungi, including *C. glabrata* (Figure 1B, asterisks), we created *C. glabrata* kinase-dead *IRE1* (*IRE1*-KD) harboring the corresponding mutations (D723N, K725N) in the Ire1 kinase domain. The growth defects of the Δ*ire1* null mutant in the presence of TM and DTT were complemented by wild-type *IRE1*, but not by *IRE1*-KD (Figure 9), indicating that the protein kinase function of Ire1 is required for the ER stress response in *C. glabrata*.

Unlike in other eukaryotes, *C. glabrata* Ire1 did not cleave *HAC1* mRNA in response to ER stress; we therefore hypothesized that the nuclease function of Ire1 would be dispensable for the ER stress response in *C. glabrata*. To test this hypothesis, we engineered *C. glabrata* nuclease-dead *IRE1* (*IRE1*-ND) containing a 10 residue (D973-Y982) internal deletion within the nuclease domain (Figure 1B). This deleted region contains three highly conserved residues (indicated by arrowheads in Figure 1B) that are the nuclease active sites of *S. cerevisiae* Ire1 [16]. A recent study has shown that deletion of the corresponding region (D1076-R1085) in *A. fumigatus* IreA abrogates its nuclease activity without an apparent effect on any nuclease-independent functions [8]. Contrary to our expectations, the Δ*ire1* mutant carrying *IRE1*-ND displayed growth defects in the presence of TM and DTT, similar to the Δ*ire1* null mutant in *C. glabrata* (Figure 9). These results suggest that both protein kinase and nuclease functions of Ire1 are required for the ER stress response in *C. glabrata*.

### The nuclease activity of Ire1 is required for degradation of ER-associated mRNAs

**Figure 10:**
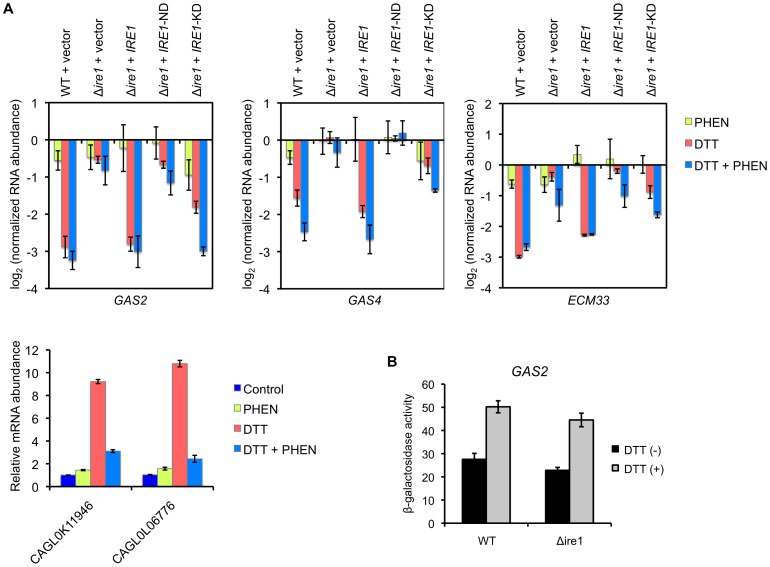
Analysis of Ire1-dependent mRNA decay in *C. glabrata*. (A) Logarithmic-phase *C. glabrata* cells were incubated in the presence and absence of 10 mM dithiothreitol (DTT) for 2 h. To examine the effects of transcription, cells were treated with the transcription inhibitor 1,10-phenanthroline (PHEN) (50 µg/ml) 5 min before DTT addition. qRT-PCR was performed as described in Materials and Methods. Expression levels of *GAS2* (CAGL0M13849g), *GAS4* (CAGL0F03883g), and *ECM33* (CAGL0M01826g) are expressed as mRNA abundance relative to the untreated control in log_2_ scale. The means and standard deviations for three independent experiments are shown. (B) β-galactosidase assay. Logarithmic-phase cells of the *C. glabrata* wild-type and Δ*ire1* strains containing pEM14-GAS2 were exposed to 3 mM DTT for 2 h. β-galactosidase activities of the *GAS2* promoter-*lacZ* fusion gene are expressed in Miller units. The means and standard deviations for three independent experiments are shown.

It has been found in higher eukaryotes that Ire1 induces degradation of mRNAs encoding proteins that are translocated into the ER lumen [62], [63], [64]. This cellular response is called regulated Ire1-dependent decay (RIDD) and represents an Xbp1-independent posttranscriptional mechanism to selectively relieve the burden of incoming proteins on the ER [63]. In our microarray analysis, a subset of genes displayed Ire1-dependent “downregulation” in response to TM (Figure S5 and Table S4). There was a strong enrichment for genes encoding GPI-anchored cell wall and membrane proteins in this cluster (Table S5). Thus, we further analyzed the expression levels of 3 representative genes, *GAS2* encoding a GPI-anchored cell wall protein that functions as β-1,3-glucanosyltransferase, *GAS4* encoding a member of the GAS family similar to *GAS2*, and *ECM33* encoding a GPI-anchored membrane protein involved in apical bud growth, by qRT-PCR using *C. glabrata* cells treated with DTT and TM. In response to the treatment with 10 mM DTT for 2 h, RNA abundance of these genes was significantly decreased in the wild-type strain compared to the results with the Δ*ire1* mutant (Figure 10A). Similar results were obtained for cells treated with 1.5 µg/ml TM in the microarray analysis (Table S4) and by a confirmatory qRT-PCR assay (data not shown). This Δ*ire1* phenotype was reversed by intact *IRE1*, but not by *IRE1*-ND (Figure 10A), indicating that the degradation of these mRNAs was dependent on the nuclease activity of Ire1. *C. glabrata IRE1*-KD harboring the simultaneous mutations D723N and K725N had moderate effects on the mRNAs decay, implying that Ire1's nuclease activity may be partially impaired by these mutations in *C. glabrata*.

Blocking transcription with 1,10-phenanthroline (PHEN) inhibited the transcriptional upregulation of CAGL0K11946g and CAGL0L06776g by DTT but had no or little effect on the mRNA dacay of *GAS2*, *GAS4* and *ECM33* (Figure 10A), indicating that the decrease in the mRNA abundance was not due to transcriptional repression. This was further supported by the observation that β-galactosidase activities of the *C. glabrata GAS2* promoter-*lacZ* fusion gene were increased, not decreased, in both the wild-type and Δ*ire1* strains in response to DTT exposure (Figure 10B). Although it is unclear why the activity of the *GAS2* promoter is increased in response to DTT, this paradoxical behavior suggest that transcriptional regulation and Ire1-dependent decay may not coordinately function in ER-stressed *C. glabrata* cells.

### Phenotypic differences between the Δ*ire1* mutant in *C. glabrata* and UPR defective mutants in other fungal species

To further investigate the roles of Ire1 in stress response in *C. glabrata*, we examined the growth of the Δ*ire1* mutants under various stress conditions. Pathogenic fungi commonly encounter a variety of adverse environmental conditions, such as low oxygen availability and nutrient depletion, in the infected host. It has been reported in *A. fumigatus* that IreA is required for growth in hypoxia and under iron-depleted conditions, which is induced by adding the Fe^2+^ chelator bathophenantroline disulphonate (BPS) to the culture medium [8]. In *C. glabrata*, the loss of Ire1 had no apparent effect on cell growth under low oxygen tension (Figure S6A) and on plates containing BPS or the bacterial siderophore desferrioxamine (DFO) (Figure S6B). *C. glabrata* cells are unable to utilize DFO, which therefore induces iron-depletion for this fungus [65]. Growth of both *C. glabrata* wild-type and Δ*ire1* strains in the presence of BPS or DFO was rescued by supplementation of the media with 1 mM FeCl_3_.

It is also reported in several pathogenic fungi, including *C. albicans*, *C. neoformans*, and *A. fumigatus*, that mutant strains lacking either Ire1 or Hac1 display growth defects in the presence of cell wall-damaging agents such as caspofungin, Congo red, and calcofluor white [6], [7], [8], [10], [11], [66]. However, in contrast to those fungi, *C. glabrata* strains lacking *IRE1*, *HAC1*, or both did not exhibit decreased tolerance to these agents at various concentrations tested (data not shown), suggesting that neither Ire1 nor Hac1 is primarily involved in cell wall stress response in *C. glabrata*.

**Figure 11:**
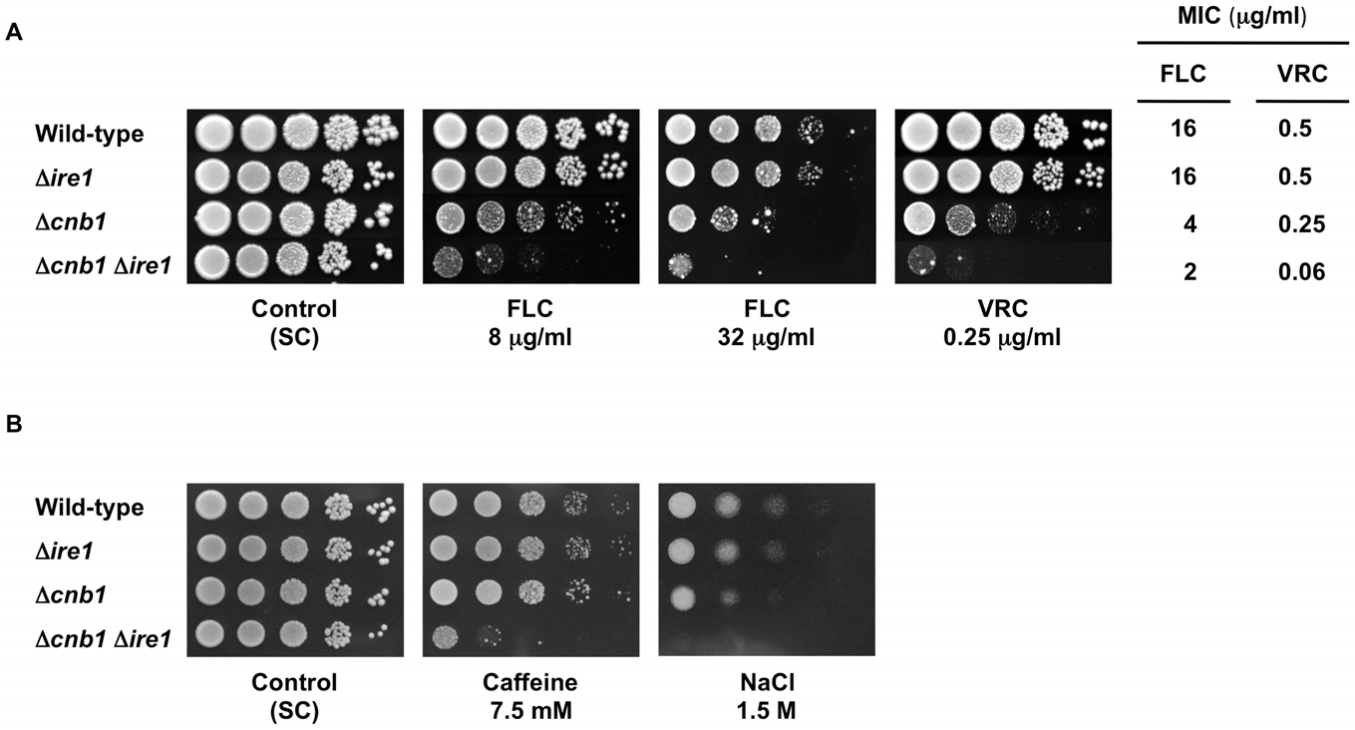
Effects of *IRE1* deletion on stress response in wild-type and Δ*cnb1* backgrounds in *C. glabrata*. (A) Ire1 plays a role in azole tolerance in *C. glabrata* when calcineurin is absent. Logarithmic-phase cells of each *C. glabrata* strain were adjusted to 2×10^7^ cells/ml, and then 5 µl of serial 10-fold dilutions were spotted onto synthetic complete (SC) plates containing either fluconazole (FLC) or voriconazole (VRC) at the indicated concentrations. Plates were incubated at 30°C for 48 h. MICs were determined by a broth microdilution method. (B) Ire1 and calcineurin serve redundant roles in cell growth under certain stress conditions in *C. glabrata*. The assay was performed as in part A. *C. glabrata* strains: Wild-type, CBS138; Δ*ire1*, TG121; Δ*cnb1*, TG161; and Δ*cnb1* Δ*ire1*, TG1612.

Importantly, disruption of Ire1/IreA confers a drastic increase in azole susceptibility in *C. neoformans* and *A. fumigatus* [7], [8]. In contrast to these findings, deletion of *IRE1* alone did not affect azole susceptibility in *C. glabrata* (Figure 11A). However, we found that the Δ*cnb1* Δ*ire1* double mutant, but not the Δ*crz1* Δ*ire1* double mutant (data not shown), was more susceptible to azole antifungals than either single mutant (Figure 11A). The results are in agreement with our previous report that calcineurin is required for azole tolerance through a Crz1-independent mechanism in *C. glabrata* [52]. Furthermore, deletion of either *CNB1* or *IRE1* alone did not affect cell growth in the presence of caffeine and NaCl, whereas the simultaneous loss of these genes resulted in severe growth defects (Figure 11B). None of these effects was observed when *IRE1* was deleted in the Δ*slt2* background (data not shown). These results suggest that calcineurin and Ire1 serve redundant roles in cell growth under certain stress conditions in *C. glabrata*. Collectively, these phenotypic analyses revealed that loss of Ire1 alone does not induce diverse phenotypes in *C. glabrata* unlike in other fungi.

### Ire1 is required for virulence in *C. glabrata*

**Figure 12:**
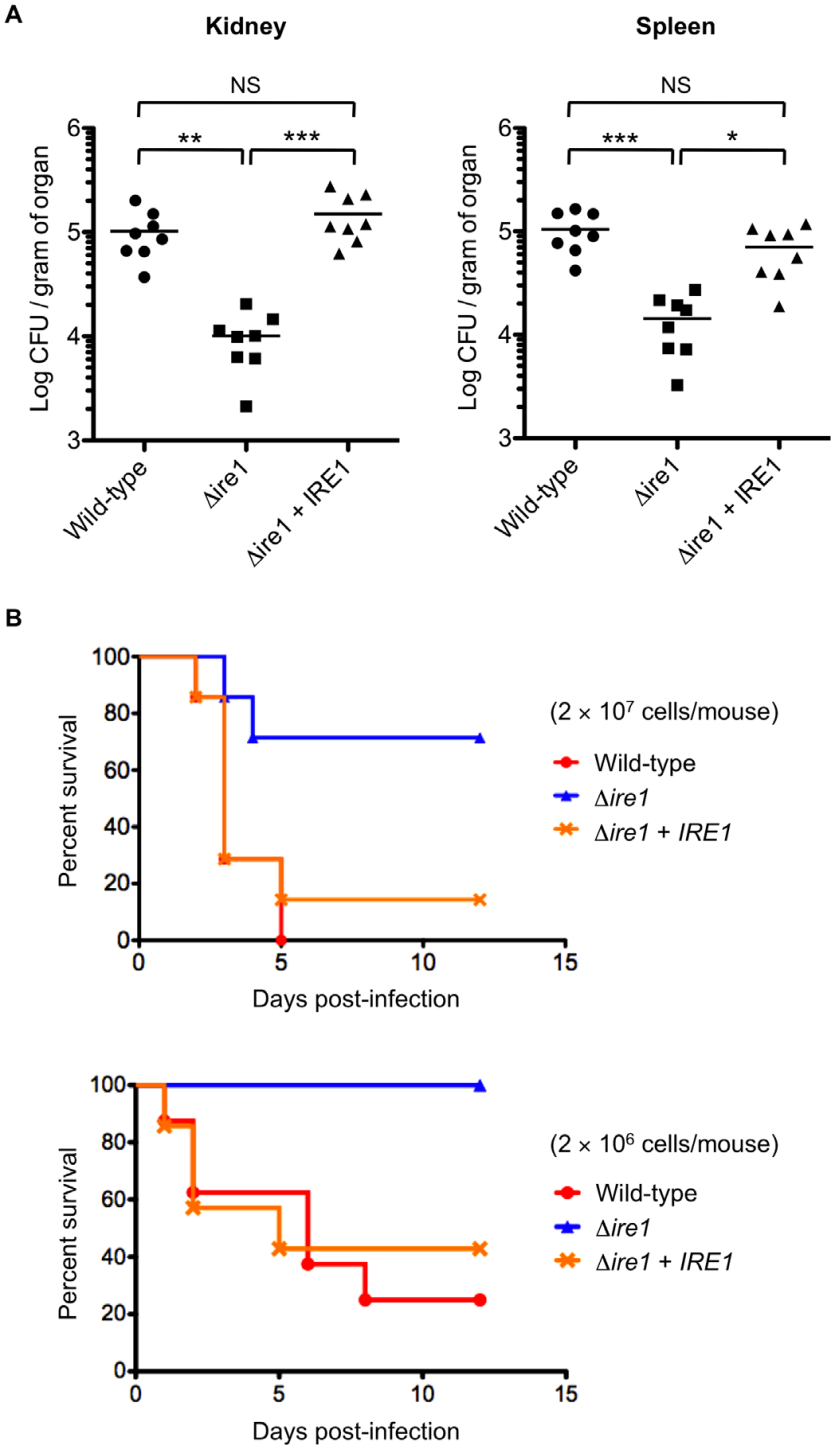
*C. glabrata* Ire1 is required for virulence in a murine model of disseminated candidiasis. (A) Groups of 8 immunocompetent mice were intravenously inoculated with 8×10^7^ cells for each *C. glabrata* strain. Bilateral kidneys and spleen were excised 7 days after injection. Appropriate dilutions of organ homogenates were plated, and the numbers of CFU were counted after 2 days of incubation at 30°C. Numbers of recovered CFU from each organ are indicated for individual mice in the scatter plots. The geometric mean is shown as a bar. Statistical analyses were performed using the Kruskal-Wallis test with Dunn's multiple comparison post-test. Asterisks indicate statistically significant differences (*: *P*<0.05; **: *P*<0.01; ***: *P*<0.001). NS indicates no significance (*P*>0.05). Representative data of two independent experiments are shown. *C. glabrata* strains: Wild-type, TG11; Δ*ire1*, TG122; and Δ*ire1*+*IRE1*, TG123. (B) Groups of 7 mice were immunosuppressed by intraperitoneal administration of cyclophosphamide (200 mg/kg/day) 72, 48, and 24 h before challenge with *C. glabrata* cells. The mice were infected intravenously with *C. glabrata* cells (2×10^7^ and 2×10^6^ cells/mouse) on Day 0 of the experiment, and survival was monitored for 12 days post-infection. Kaplan-Meier curves were created and compared with the log rank (Mantel-Cox) test. Upper panel (2×10^7^ cells/mouse): *P* = 0.0078 for wild-type vs. Δ*ire1*, *P* = 0.0284 for Δ*ire1* vs. Δ*ire1*+*IRE1*, and *P* = 0.6768 for wild-type vs. Δ*ire1*+*IRE1*; lower panel (2×10^6^ cells/mouse): *P* = 0.0043 for wild-type vs. Δ*ire1*, *P* = 0.0222 for Δ*ire1* vs. Δ*ire1*+*IRE1*, and *P* = 0.7440 for wild-type vs. Δ*ire1*+*IRE1*.

We have previously demonstrated in *C. glabrata* that calcineurin and Slt2 are required for virulence in murine models of disseminated candidiasis [52], [67], but the involvement of Ire1 in *C. glabrata* virulence has not been examined. The *C. neoformans* Δ*ire1* and *A. fumigatus* Δ*ireA* mutants are avirulent in murine models of systemic cryptococcosis and invasive aspergillosis, respectively, which can be explained primarily by their growth defects at 37°C [7], [8]. In *C. glabrata*, the loss of Ire1 did not affect cell growth at 37°C (generation time of the wild-type and Δ*ire1* strains in Yeast-peptone-dextrose (YPD) broth was 75 and 78 min, respectively). Therefore, we compared the virulence of the *C. glabrata* wild-type, Δ*ire1*, and *IRE1*-complemented strains in a mouse model of disseminated candidiasis. Immunocompetent mice infected with the Δ*ire1* mutant displayed significantly reduced fungal burden in both kidney and spleen relative to those infected with the wild-type and Ire1-complemented strains (Figure 12A). To evaluate mortality of mice with disseminated candidiasis, mice were immunosuppressed by cyclophosphamide prior to *C. glabrata* infection. Consistent with the results of organ fungal burden, mice infected with the wild-type and *IRE1*-complemented strains exhibited higher mortality rates than mice infected with the Δ*ire1* mutant (Figure 12B). These results suggest that Ire1 is important for virulence in *C. glabrata*.

## Discussion

According to the current evidence, the UPR regulated by Ire1-Hac1 signaling is highly conserved and is a key pathway to cope with ER stress in eukaryotes [2], [12]. However, the present study demonstrates the lack of this pathway and the development of alternative mechanisms for the ER stress response in *C. glabrata*. The gene CAGL0K12540g, named *C. glabrata HAC1*, seems to be the sole *HAC1* ortholog in *C. glabrata*, since it is the only gene showing sequence similarity and conserved synteny to *S. cerevisiae HAC1*. In addition, *C. glabrata* Hac1 was able to complement *S. cerevisiae* Hac1 functions. However, surprisingly, *C. glabrata* Hac1 did not necessitate Ire1-mediated splicing of mRNA before translation, and thus could recover the growth of the *S. cerevisiae* Δ*ire1* mutant in the presence of ER stress. A recent study has comprehensively characterized the RNA structures of the Hac1/Xbp1 orthologs in various eukaryotic species [36]. In *C. glabrata HAC1*, although the overall RNA structure is maintained, the predicted intron (379 nucleotides) is much longer than those in other species and has mutations in the Ire1 recognition motifs at both splice sites, suggesting that the Ire1-dependent unconventional splicing mechanism may not be present in *C. glabrata* [36]. This has been experimentally confirmed in our studies: the heterologous expression of *C. glabrata HAC1* complemented *S. cerevisiae* Hac1 functions without splicing, and *C. glabrata HAC1* mRNA was not spliced by Ire1 even in ER-stressed *C. glabrata* cells. Furthermore, Hooks and Griffiths-Jones [36] revealed that the non-canonical Hac1/Xbp1 intron structure is not conserved in 28 out of 156 eukaryotic species searched, including several *Candida* species such as *Candida parapsilosis*, *Candida lusitaniae*, and *Candida guilliermondii*, despite the fact that the *HAC1* ORFs are intact in these *Candida* species. These findings suggest that these species may have lost the unconventional splicing mechanism, but instead acquired an alternative mechanism to regulate the UPR. Future studies are warranted to determine whether Hac1 is involved in the ER stress response in these species.

Although some Hac1-independent functions of Ire1 have been reported in some eukaryotic species [2], [7], [8], [63], mutants lacking Hac1 or its homolog always phenocopy Δ*ire1* strains with respect to growth defects in the presence of ER stress-inducing agents (e.g. TM and DTT) in all eukaryotic species tested so far, and the UPR mediated by Ire1-Hac1 signaling has been believed to be a phylogenetically conserved pathway in eukaryotes [2], [68], [69]. However, our study demonstrated that *C. glabrata* Ire1 plays a role in the ER stress response in a Hac1-independent manner. Exploration of potential Ire1 targets in this fungus by a genome-wide analysis will be of interest in future investigations (e.g. RNA-seq analysis of ER-stressed and unstressed cells of wild-type and Δ*ire1* strains to detect Ire1-dependent spliced RNAs). The results of our gene expression assays suggest that Ire1 does not mediate transcriptional response to ER stress in *C. glabrata*, raising the question of how Ire1 is involved in the ER stress response in this fungus.

In metazoans, ER stress triggers two distinct outputs of Ire1's nuclease activity, *XBP1* splicing and RIDD [69], [70]. The latter is an Xbp1-independent pathway that selectively degrades a small subset of ER-associated mRNAs and remodels the repertoire of proteins translated in ER-stressed cells [63], [64]. This cellular response is predicted to reduce the ER load by limiting protein influx and unfolded protein load into the ER lumen. The RIDD pathway was first uncovered in *D. melanogaster* [64], and later, its conservation was confirmed in mammalian cells [62], [63], but has yet to be fully determined in fungi. Interestingly, in *S. cerevisiae*, RIDD does not occur [63], [70], but the classic Ire1-Hac1 UPR mediates downregulation of some mRNAs encoding membrane proteins [64], [71]. Here, we have demonstrated that mRNA abundance of a subset of genes encoding GPI-anchored cell wall and membrane proteins was diminished during the response to ER stress in the wild-type strain dependent on the nuclease activity of Ire1. There was no overlap between these repressed *C. glabrata* genes and the previously identified *S. cerevisiae* gene that were transcriptionally downregulated by the UPR in response to TM [71]. It is thought that RIDD targets are nicked by the Ire1 endoribonuclease at sites that do not display an identifiable consensus sequence, in contrast to Ire1-mediated splicing of *HAC1*/*XBP1* mRNA at the highly conserved splice junctions [69]. As excessive activation of RIDD seems to be detrimental to cell integrity [62], reducing the load of proteins entering the ER must be balanced with the need to sustain synthesis of essential proteins. In contrast to Ire1 in metazoans, *C. glabrata* Ire1 may not need to switch between specific and nonspecific modes of cleavage. We are currently investigating how the RIDD activity is regulated in this fungus.

Although the only known substrate of the Ire1 kinase is Ire1 itself in *S. cerevisiae* [30], [31], we also addressed the possibility that Ire1 may integrate with other signaling pathways in *C. glabrata*. In *S. cerevisiae*, calcineurin and Slt2 are also involved in the ER stress response through different mechanisms [48], [49], [50], [51]. Slt2 plays a role in the ER stress surveillance (ERSU) pathway that ensures transmission of only functional ER to daughter cells during cell division [72]. Upon ER stress, the ERSU pathway delays ER inheritance and cytokinesis to prevent death of both mother and daughter cells. Calcineurin mediates the calcium cell survival pathway by regulating intracellular Ca^2+^ homeostasis. TM increases Ca^2+^ uptake by stimulating the Cch1-Mid1 high affinity Ca^2+^ channel, while calcineurin dephosphorylates the Cch1 subunit of the channel to inhibit Ca^2+^ influx and prevents nonapoptotic cell death in *S. cerevisiae* [48], [51]. In *C. glabrata*, loss of either calcineurin or Slt2 resulted in decreased tolerance to ER stress, and the additional deletion of *IRE1* in the Δ*cnb1* and the Δ*slt2* backgrounds had synthetic effects on viability loss in the presence of TM. The results suggest that Ire1 functions in parallel with the calcineurin and Slt2 MAPK pathways to cope with ER stress in *C. glabrata*, consistent with previous findings in *S. cerevisiae* [48], [49], [50]. However, the possibility that Ire1 might interact with other signaling pathways has not been completely ruled out. Whether the Ire1 kinase domain has an effector other than Ire1 itself remains one of the major unsolved questions in this field [69].

The term UPR is derived from a cellular response to chemicals that interfere with proper protein folding (e.g. TM and DTT), but it is now known that the signaling pathway is involved in various stress responses from yeast to humans [73], [74]. Indeed, downstream targets of the UPR include not only ER chaperones, but also genes involved in diverse functions, including protein trafficking and quality control, lipid and sterol metabolism, heme biosynthesis, and cell wall biogenesis in *S. cerevisiae* [20]. To extend our understanding of Ire1 functions in *C. glabrata*, phenotypes of the Δ*ire1* mutant were investigated in various stress conditions in view of clinical significance. The UPR is critical for azole tolerance in *C. neoformans* and *A. fumigatus*, and thus loss of either Ire1/IreA or Hxl1/HacA leads to increased susceptibility to azole antifungals in these species [7], [8], [10]. In contrast, loss of Ire1, Hac1, or both did not affect azole tolerance in *C. glabrata* (Figure 11A and data not shown), further supporting the idea that the classic UPR mechanism regulated by Ire1-Hac1 signaling is not conserved in this fungus. However, the additional deletion of *IRE1* in the Δ*cnb1* background increased azole susceptibility in *C. glabrata*. One possible explanation is that azole antifungals induce severe ER stress in calcineurin-defective *C. glabrata* cells, which therefore require Ire1 to survive this stress condition.

Previous studies in several yeasts and filamentous fungi have demonstrated that the UPR plays a role in maintaining cell wall integrity, and that mutants lacking Ire1 or Hac1 exhibit decreased tolerance to a variety of cell wall perturbing agents [6], [7], [8], [10], [11], [66]. TM also induces cell wall stress, thereby explaining why certain genes implicated in cell wall biogenesis are upregulated during the UPR in TM-treated *S. cerevisiae* and *C. albicans* cells [11], [20], [75]. In contrast to other fungi investigated to date, the loss of Ire1 alone did not confer a cell wall-defective phenotype, and upregulation of some cell wall-related genes in response to TM exposure was mainly dependent on calcineurin, but not on Ire1, in *C. glabrata* (Table S2). These results provide additional evidence that there are diversities in Ire1-dependent stress response mechanisms between *C. glabrata* and other fungal species. In addition, Δ*ire1*/*ireA* mutants of *C. neoformans* and/or *A. fumigatus* exhibit growth defects under various environmental conditions, such as growth temperature at 37°C, hypoxia, and iron limitation, which are commonly encountered by pathogenic fungi in the infected host [7], [8]. However, none of these phenotypes were observed in the *C. glabrata* Δ*ire1* mutant. In agreement with our proposal that the canonical Ire1-Hac1 UPR has been lost in *C. glabrata*, phenotypes of the *C. glabrata* Δ*ire1* mutant were confined relative to those of UPR-defective mutants in other fungi where the UPR is involved in various stress responses.

Diversity in the transcriptional induction system in response to ER stress has developed during evolution [68]. For instance, the Ire1-Hac1 pathway is required for upregulation of *KAR2* in yeast while the pathway is dispensable for induction of the Kar2 homolog GRP78/BiP in mammalian cells [17], [76]. In *C. glabrata*, the majority of genes, including *KAR2*, induced in response to ER stress were dependent on the calcineurin-Crz1 pathway, but not on Ire1 signaling. In addition, several lines of evidence described above demonstrate that *C. glabrata* has lost the classic Ire1-Hac1 UPR, but instead possesses an alternative mechanism, RIDD, like metazoans. To our knowledge, these unusual characteristics have been demonstrated for the first time in any eukaryote, and perhaps they were developed in *C. glabrata* after its divergence from *S. cerevisiae* due to its ecological niche. Our results provide novel insights into ER stress response mechanisms as well as fundamental information for evolutionary biology to further understand how eukaryotic cells have developed ER quality control systems.

## Materials and Methods

### Ethics statement

All animal experiments were performed in full compliance with the Guide for the Care and Use of Laboratory Animals (National Research Council, National Academy Press, Washington DC, 2011) and all of the institutional regulations and guidelines for animal experimentation after pertinent review and approval by the Institutional Animal Care and Use Committee of Nagasaki University under protocol number 0904130747.

### Strains and culture conditions

The *C. glabrata* and *S. cerevisiae* strains used in this study are listed in Table S6. Cells were routinely propagated at 30°C in YPD, synthetic complete (SC) medium lacking appropriate amino acids, or synthetic defined (SD) medium [77], unless otherwise indicated. The nitrogen starvation medium, SD-N, consists of 0.17% yeast nitrogen base without amino acids and ammonium sulfate (BD Biosciences, Franklin Lakes, NJ) plus 2% glucose as described previously [78]. The Anaeropack system (Mitsubishi Gas Chemical Company Inc, Tokyo, Japan) was used for cultures under conditions of low oxygen tension (hypoxic). To induce iron-depleted conditions, the Fe^2+^ chelator BPS (MP Biomedicals, Solon, OH) or the bacterial siderophore DFO (EMD Chemicals, San Diego, CA) were added to SC media at final concentrations of 20 and 50 µM, respectively.

### Plasmid and strain construction

The primers and plasmids used in this study are listed in Tables S7 and S8, respectively. Transformation of *C. glabrata* and *S. cerevisiae* was performed using a lithium acetate protocol as described previously [79]. *C. glabrata* deletion mutants were constructed using a one-step PCR-based technique as described previously [67]. Briefly, a deletion construct was amplified from pBSK-TRP or pBSK-HIS using primers tagged with the 100 bp sequences homologous to the flanking regions of the target ORF. *C. glabrata* parent strains were transformed with the deletion construct, and the resulting transformants were selected by tryptophan or histidine prototrophy. PCR and Southern blotting were performed to verify that the desired homologous recombination occurred at the target locus.

To generate complementation plasmids, *C. glabrata* and *S. cerevisiae* genes were amplified from the genomic DNA of CBS138 [80] and BY4742 [81], respectively. Procedures for each plasmid construction are summarized in Table S8. The spliced form of *S. cerevisiae HAC1* mRNA was obtained from BY4742 cells treated with 1.5 µg/ml TM for 3 h. The extracted RNA was then reverse-transcribed using a QuantiTect Reverse Transcription kit (Qiagen, Valencia, CA), and the resulting cDNA was used as a template to amplify the mature *S. cerevisiae HAC1*, *ScHAC1^i^* (“*i*” for induced).

The plasmid pCgACT-PIRE was used as the template to generate pCgACT-PIRE-KD and pCgACT-PIRE-ND. pCgACT-PIRE-KD was generated by mutating two residues, D723 and K725, in the kinase domain of *C. glabrata* Ire1 to asparagines using the KOD-Plus-Mutagenesis Kit (Toyobo, Osaka, Japan), and mutagenic primers CgIRE1-mut-F2171 and CgIRE1-mut-R2170. Similarly, pCgACT-PIRE-ND was created by introducing a 10 residue (D973-Y982) internal deletion within the nuclease domain of *C. glabrata* Ire1 using mutagenic primers, CgIRE1-F2947 and CgIRE1-R2916. All of the plasmids constructed using PCR products were verified by sequencing before use.

### Drug susceptibility assay

Spot dilution tests and time-kill assays were performed as described previously [52]. Voriconazole was kindly provided by Pfizer (New York, NY). Other drugs were purchased from Sigma (St Louis, MO). TM, voriconazole, and caffeine were dissolved in dimethyl sulfoxide (DMSO), and others were dissolved in distilled water. DMSO alone did not interfere with cell growth at the final concentrations used in this study. MICs of azole antifungals were determined by a broth microdilution test using a commercially prepared colorimetric microdilution panel (ASTY; Kyokuto Pharmaceutical Industrial Co., Ltd.) [82]. All sensitivity tests were performed on at least two separate occasions to ensure reproducibility.

### Analysis of *HAC1* mRNA splicing by RT-PCR and Northern blotting

*HAC1* mRNA splicing was analyzed using a modification of a reported protocol [66]. Logarithmic-phase *S. cerevisiae* cells grown in SC medium lacking leucine were treated with 1.5 µg/ml TM or 5 mM DTT for 3 h. Logarithmic-phase *C. glabrata* cells grown in SC medium were treated with either TM (5 and 10 µg/ml) or DTT (5 and 10 mM) for 1 and 3 h. These cells were harvested, and total RNA was extracted using a FastRNA Red Kit (Qbiogene, Carlsbad, CA) according to the manufacturer's instructions. cDNA was synthesized from 1 µg of total RNA using a QuantiTect Reverse Transcription kit (Qiagen) in a final volume of 20 µl, and 5 µl of resulting cDNA were then used as the template for individual PCR. The amplified PCR products were analyzed by both electrophoresis on a 1% agarose gel and DNA sequencing. For Northern blot analysis, twenty micrograms total RNA was separated on a 2% agarose gel and transferred to a Hybond-N^+^ membrane (GE Healthcare, Buckinghamshire, UK), which was incubated in PerfectHyb Hybridization Solution (Toyobo) with probes directed against the 5′ region of the *HAC1* ORF. All probes were generated by PCR with primers listed in Table S7 and were labeled with [α-^32^P]dCTP using a Random Primer DNA Labeling Kit Ver.2.0 (Takara Bio Inc., Shiga, Japan). Images were obtained using Image Reader FLA-5000 (FUJIFILM Corporation, Tokyo, Japan).

### qRT-PCR

Total RNA extraction and cDNA synthesis were performed as described above, and 3 µl of resulting cDNA were used as the template for individual PCR with a QuantiTect SYBR Green PCR kit (Qiagen). qRT-PCR was carried out in triplicate in a 96-well plate format, using a 7500 Real-Time PCR System (Applied Biosystems, Foster City, CA). The mRNA abundance of the target genes was normalized to 18S rRNA (CAGL0L13398r). The qRT-PCR assays were repeated at least twice on independent occasions.

### Microarray analysis

*C. glabrata* cells were grown in SC medium to exponential phase (OD600 = 0.8) and exposed to 1.5 µg/ml TM at 30°C. Total RNAs were extracted using the FastRNA Red Kit (Qbiogene). The quality of RNA was checked with a RNA 6000 Nano Kit and Agilent 2100 Bioanalyzer. Double-stranded cDNA was synthesized using the Invitrogen SuperScript Double-Stranded cDNA Synthesis Kit and oligo (dT) primers. The resulting cDNA samples were labeled with Cy3 using the NimbleGen One-Color DNA Labeling Kit and subsequently hybridized to a custom-made 4×72 K *C. glabrata* array (Roche NimbleGen, Tokyo, Japan) wherein each chip measures the expression levels of 5,217 genes from *C. glabrata* CBS138 with six 60-mer-probe pairs per gene, with two-fold technical redundancy. The arrays were washed using the NimbleGen Wash Buffer Kit and scanned with a NimbleGen MS 200 Microarray Scanner. Data were quantified using the NimbleScan v2.6 software and normalized as described previously [83], [84]. Hierarchical clustering was performed using Gene Cluster 3.0 and visualized using Java TreeView ver. 1.1.5r2. Gene Ontology (GO) enrichment was searched using the Candida Genome Database GO term finder (http://www.candidagenome.org/cgi-bin/GO/goTermFinder) with default parameters [85].

### β-galactosidase assay

A 699-bp DNA fragment containing the 5′ untranslated region (UTR) and the first 17 codons of the *C. glabrata GAS2* gene was amplified from the genomic DNA of CBS138 and fused to the *lacZ* reporter in pEM14 [86] to construct pEM14-GAS2 as described in Table S8. Logarithmic-phase cells of the *C. glabrata* wild-type and Δ*ire1* strains containing pEM14-GAS2 were grown in SC-ura broth, adjusted to 1×10^7^ cells/ml, and then incubated in the presence and absence of 3 mM DTT for 2 h. β-galactosidase assay was performed as described previously [59]. The cell cultures were harvested and washed twice with ice-cold phosphate-buffered saline. Cells (100 µl) were re-suspended in 300 µl Reporter Lysis Buffer (Promega, Madison, WI) containing 5 µl Protease Inhibitor Cocktail (Sigma). Cell extracts were prepared using acid-washed glass beads (Sigma) and cleared by centrifugation at 14,000× *g* for 30 min at 4°C. Protein concentrations were determined by the Bio-Rad Protein Assay (Bio-Rad, Richmond, CA) using bovine serum albumin as a standard. β-galactosidase activities were measured using the β-Galactosidase Enzyme Assay System (Promega) according to the manufacturer's instructions and calculated in Miller units (nmoles/min/mg of protein) at 37°C [87]. All assays were performed in triplicate on separate days.

### Virulence assay

Animal experiments were performed as described previously [52], [67]. Briefly, to prepare cells for injection, logarithmic-phase *C. glabrata* cells were harvested, washed, resuspended in sterile saline, and adjusted to 4×10^8^ cells/ml after counting the number of cells using a hemocytometer. The actual CFU in the inocula were confirmed by plating serial dilutions of cell suspension on YPD plates. Groups of 8 female, 8-week-old, BALB/c mice (Charles River Laboratories Japan Inc., Japan) were injected with 0.2 ml of the *C. glabrata* cell suspension via the lateral tail vein. The mice were euthanized 7 days after injection and the spleen and bilateral kidneys were then excised. No mice died before euthanasia in this experiment. Appropriate dilutions of organ homogenates were plated on YPD plates. Colonies were counted after 2 days of incubation at 30°C and the CFUs per organ were calculated. Statistical analyses were performed using the Kruskal-Wallis test with Dunn's multiple comparison post-test (GraphPad Prism 5, La Jolla, CA).

To examine mortality of mice with disseminated candidiasis due to *C. glabrata*, groups of 7 female BALB/c mice (20–23 g body weight, mean 20.6–20.9 g for each group) were immunosuppressed by intraperitoneal administration of cyclophosphamide at a concentration of 200 mg/kg/day on days −3, −2, and −1 of infection, and housed under sterile conditions. The mice were inoculated intravenously with 0.2 ml of *C. glabrata* cell suspensions (1×10^8^ and 1×10^7^ cells/ml) on day 0. Checks were made for dead or moribund mice in a blinded fashion twice daily over a period of 16 days. A group of 4 immunosuppressed mice was injected with sterile saline instead of *C. glabrata* cell suspension, and no mice died due to immunosuppression only. Survival was plotted on a Kaplan-Meier curve for each *C. glabrata* strain, and the log rank (Mantel-Cox) test was used for pairwise comparison of percent survival (GraphPad Prism 5). A *P* value of <0.05 was considered statistically significant. These animal experiments were conducted on two separate occasions to ensure reproducibility.

### Accession numbers for genes and proteins mentioned in the text (NCBI Entrez Gene ID or GenBank accession number)

*C. glabrata*: 18S rRNA (9488051); *ACT1* (2890423); *CAD1/YAP2* (2887750); CAGL0A04081g (2886450); CAGL0F04829g (2887774); CAGL0I07249g (2889193); CAGL0I08591g (2888926); CAGL0K11946g (2889962); CAGL0L06776g (2890816); CAGL0M04191g (2891538); CAGL0M12320g (2891428); *CNB1* (2890566); *CRZ1* (2891693); *DER1* (2891627); *ECM33* (2891194); *FPR2* (2888506); *GAS2* (2891237); *GAS4* (2887679); *GCN4* (2890908); *HAC1* (2890416); *HIS3* (2890838); *IRE1* (2887705); *KAR2* (2887030); *PDI1* (2886594); *SKO1* (2889579); *SLT2* (2889880); *TRP1* (2886909); *YAP1* (2888604); *YAP3*/CAGL0K02585g (2890264); *YAP3*/CAGL0M10087g (2891349); *YAP6* (2891289); *YAP7* (2887954).

*S. cerevisiae*: *ACT1* (850504); *ADH1* (854068); *CCH1* (853131); *DER1* (852500); *FPR2* (852131); *HAC1* (850513); *IRE1* (856478); *KAR2* (853418); *MID1* (855425); *PDI1* (850314); *PGK1* (850370); *SLT2* (856425).

Others: *A. fumigatus* HacA/Hac1 (3506096); *A. fumigatus* IreA/Ire1 (AEQ59230); *A. nidurans* HacA (CBF87535); *C. albicans* Hac1 (3639758); *C. albicans* Ire1 (3640823); *C. elegans* Xbp1 (175541); *C. neoformans* Hxl1 (3255200); *C. neoformans* Ire1 (3255994); *D. melanogaster* Xbp1 (44226); *H. sapiens* Xbp1 (7494); *P. pastoris HAC1* (8199414); *T. reesei HAC1* (AJ413272); *Y. lipolytica HAC1* (2906724).

## Supporting Information

Figure S1 Comparison of cell growth in the presence of ER stress between various fungal species. Logarithmic-phase cells of each fungal strain were harvested after appropriate incubation in YPD broth. Cell concentration was adjusted with optical density at 600 nm, and then 5 µl of serial 10-fold dilutions were spotted onto YPD or synthetic complete (SC) plates containing either tunicamycin (TM) or dithiothreitol (DTT) at the indicated concentrations. Plates were incubated at 30°C for 48 h.
(TIF)

Figure S2 Synteny relationships of the *IRE1* and *HAC1* loci between *S. cerevisiae* and *C. glabrata*. (A) Schematic representation of the *C. glabrata IRE1* (CAGL0F03245g) locus compared to syntenic regions of the *S. cerevisiae* genome. Colored arrows indicate orthologs in the two species. White arrows indicate *S. cerevisiae* genes of which orthologs are not located around *IRE1* in the *C. glabrata* genome. (B) Synteny analysis of the *C. glabrata HAC1* (CAGL0K12540g) and *S. cerevisiae HAC1* loci.
(TIF)

Figure S3 Disruption of *HAC1* in *C. glabrata*. The entire open reading frame (ORF) of *HAC1* was replaced with the deletion construct containing a *TRP1* marker. Southern blot analysis of ClaI-digested genomic DNA using a *HAC1* probe, which was designed within the *HAC1* ORF, identified the predicted 4.3 kb band in the parent strain 2001HT (Δ*his3* Δ*trp1*) but no band in the Δ*hac1* mutant TG141 (Δ*hac1*::*TRP1* Δ*his3*). A second Southern blot analysis using a *TRP1* probe confirmed that the desired homologous recombination had occurred at the *HAC1* locus without ectopic integration of the deletion construct in the mutant.
(TIF)

Figure S4 qRT-PCR analysis to validate expression profiles of representative UPR target genes in the presence of tunicamycin (TM) and dithiothreitol (DTT). *C. glabrata* wild-type (CBS138) cells were treated with TM or DTT at the indicated concentrations for 1 and 3 h. Expression levels of *KAR2* (A) and other known UPR targets (B) were examined by qRT-PCR as described in Materials and Methods. The means and standard deviations for three independent experiments are shown.
(TIF)

Figure S5 Genome-wide gene expression profiles in response to tunicamycin (TM) exposure for 0.5 h. Hierarchical clustering of genes whose expression levels were changed more than 1.5-fold after treatment with 1.5 µg/ml TM for 0.5 h. Genes were clustered with average linkage. *C. glabrata* strains: WT, 2001T; Δ*cnb1*, TG161; Δ*crz1*, TG171; Δ*slt2*, TG151; and Δ*ire1*, TG121.
(TIF)

Figure S6 Ire1 is dispensable for growth under conditions of hypoxia and iron depletion in *C. glabrata*. (A) Logarithmic-phase cells of each *C. glabrata* strain were adjusted to 2×10^7^ cells/ml, and then 5 µl of serial 10-fold dilutions were spotted onto YPD plates. One plate was incubated under normal (aerobic) condition for 44 h, and the other plate was incubated under conditions of low oxygen tension (hypoxic) for 68 h. (B) Serial dilutions of *C. glabrata* cells were prepared as described above and spotted onto synthetic complete (SC) plates containing either 20 µM bathophenantroline disulphonate (BPS) or 50 µM desferrioxamine (DFO) in the presence and absence of 1 mM ferric chloride (FeCl_3_). Plates were incubated at 30°C for 24 h.
(TIF)

Table S1 Putative *C. glabrata* bZIP transcription factors identified by a BLASTp search using the bZIP domain sequences of *S. cerevisiae* Hac1, *H. sapiens* Xbp1, and *C. neoformans* Hxl1 as queries.
(DOC)

Table S2 List of genes whose expression levels were altered by tunicamycin treatment for 3 h.
(XLSX)

Table S3 Classification of the putative Slt2-repressed genes.
(XLSX)

Table S4 List of genes whose expression levels were altered by tunicamycin treatment for 0.5 h.
(XLSX)

Table S5 Classification of the putative Ire1-repressed genes.
(XLSX)

Table S6 Strains used in this study.
(DOC)

Table S7 Primers used in this study.
(DOC)

Table S8 Plasmids used in this study.
(DOC)
