# Supplementary figures and images for: Dissection of Ire1 Functions Reveals Stress Response Mechanisms Uniquely Evolved in Candida glabrata
Source: PLoS Pathog. 2013 Jan 31;9(1):e1003160. doi: 10.1371/journal.ppat.1003160 (PMC3561209; doi:10.1371/journal.ppat.1003160)

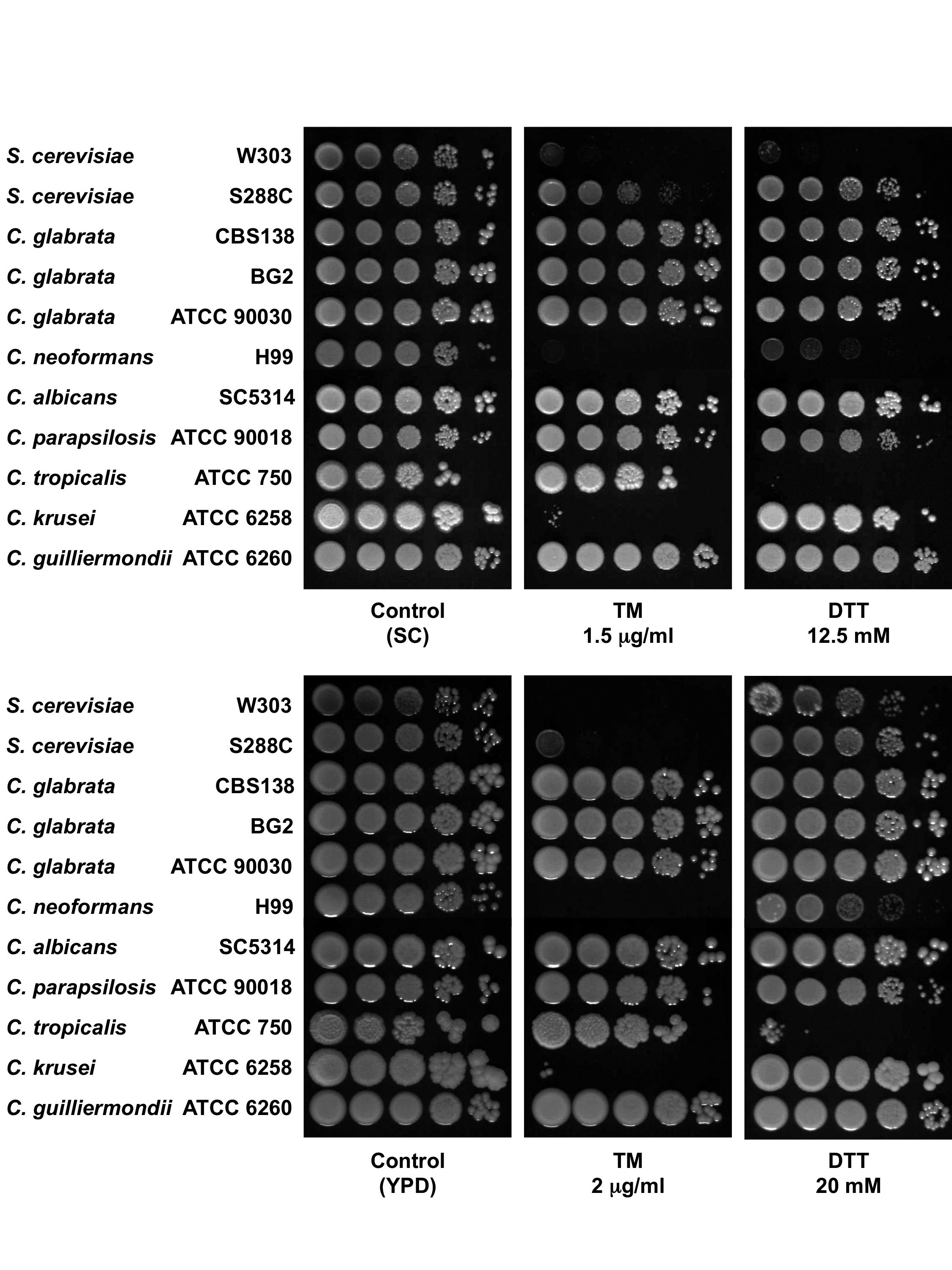

Supplement: Figure S1 — Comparison of cell growth in the presence of ER stress between various fungal species. Logarithmic-phase cells of each fungal strain were harvested after appropriate incubation in YPD broth. Cell concentration was adjusted with optical density at 600 nm, and then 5 µl of serial 10-fold dilutions were spotted onto YPD or synthetic complete (SC) plates containing either tunicamycin (TM) or dithiothreitol (DTT) at the indicated concentrations. Plates were incubated at 30°C for 48 h. (TIF) [file ppat.1003160.s001.tif]

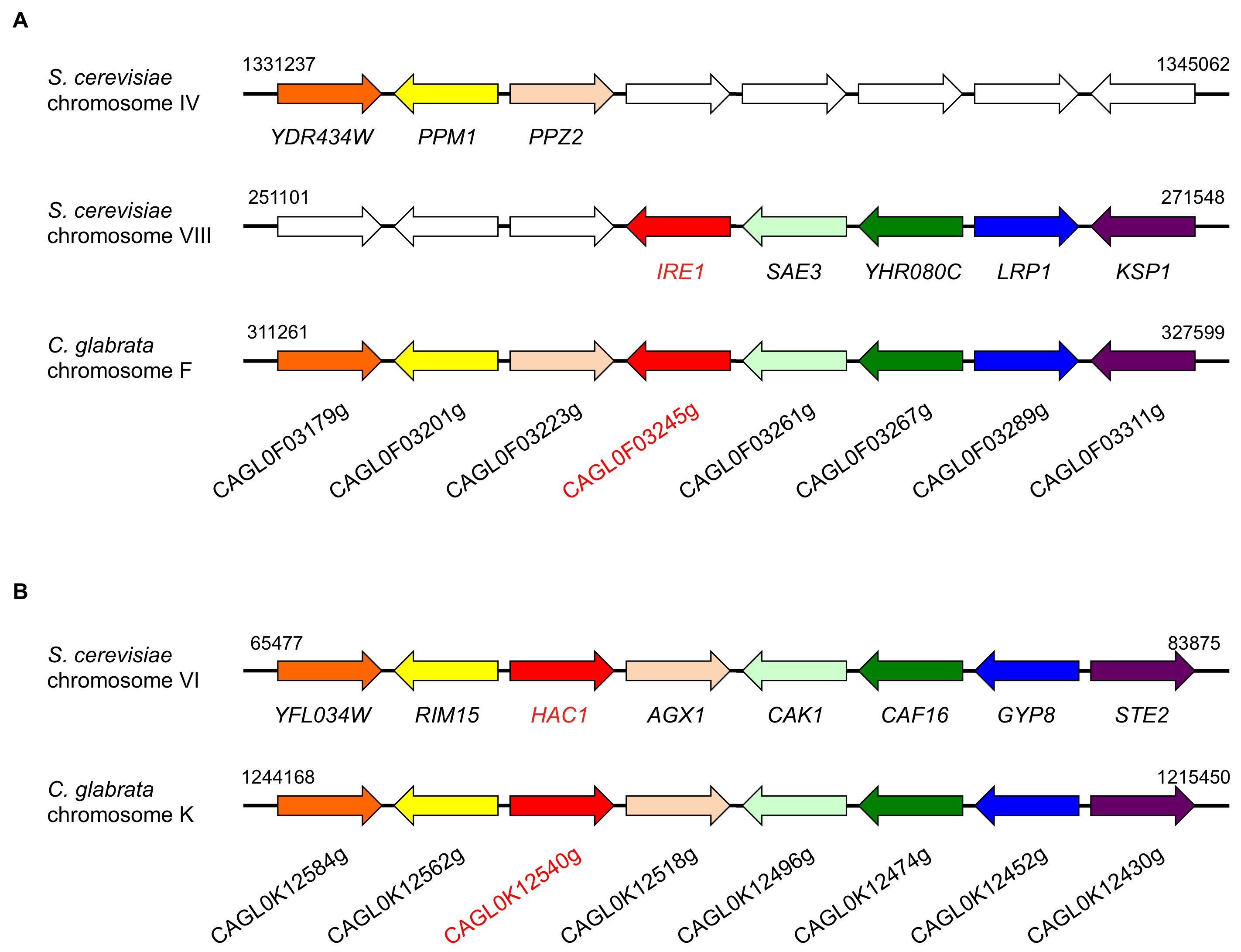

Supplement: Figure S2 — Synteny relationships of the IRE1 and HAC1 loci between S. cerevisiae and C. glabrata. (A) Schematic representation of the C. glabrata IRE1 (CAGL0F03245g) locus compared to syntenic regions of the S. cerevisiae genome. Colored arrows indicate orthologs in the two species. White arrows indicate S. cerevisiae genes of which orthologs are not located around IRE1 in the C. glabrata genome. (B) Synteny analysis of the C. glabrata HAC1 (CAGL0K12540g) and S. cerevisiae HAC1 loci. (TIF) [file ppat.1003160.s002.tif]

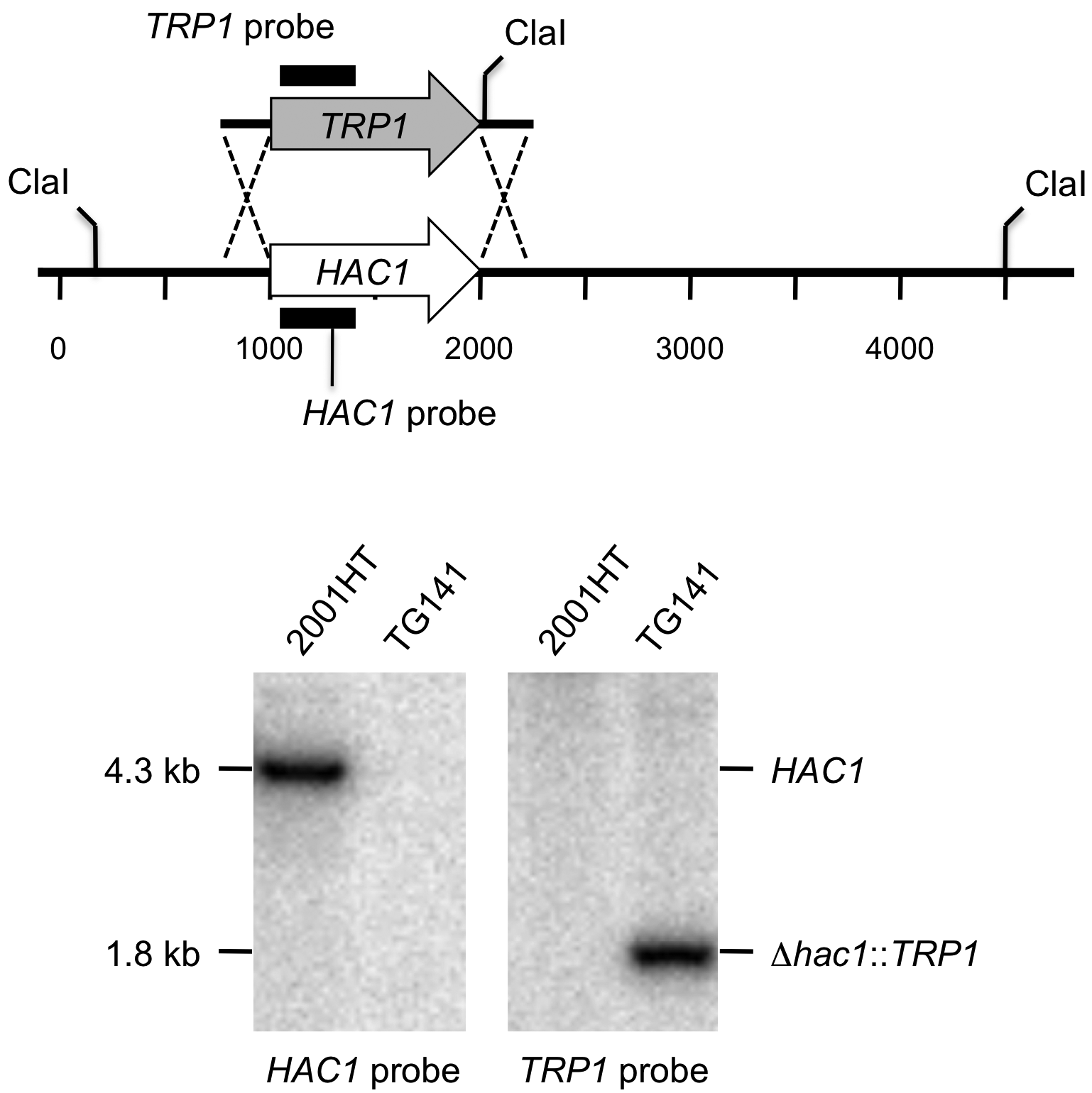

Supplement: Figure S3 — Disruption of HAC1 in C. glabrata. The entire open reading frame (ORF) of HAC1 was replaced with the deletion construct containing a TRP1 marker. Southern blot analysis of ClaI-digested genomic DNA using a HAC1 probe, which was designed within the HAC1 ORF, identified the predicted 4.3 kb band in the parent strain 2001HT (Δhis3 Δtrp1) but no band in the Δhac1 mutant TG141 (Δhac1::TRP1 Δhis3). A second Southern blot analysis using a TRP1 probe confirmed that the desired homologous recombination had occurred at the HAC1 locus without ectopic integration of the deletion construct in the mutant. (TIF) [file ppat.1003160.s003.tif]

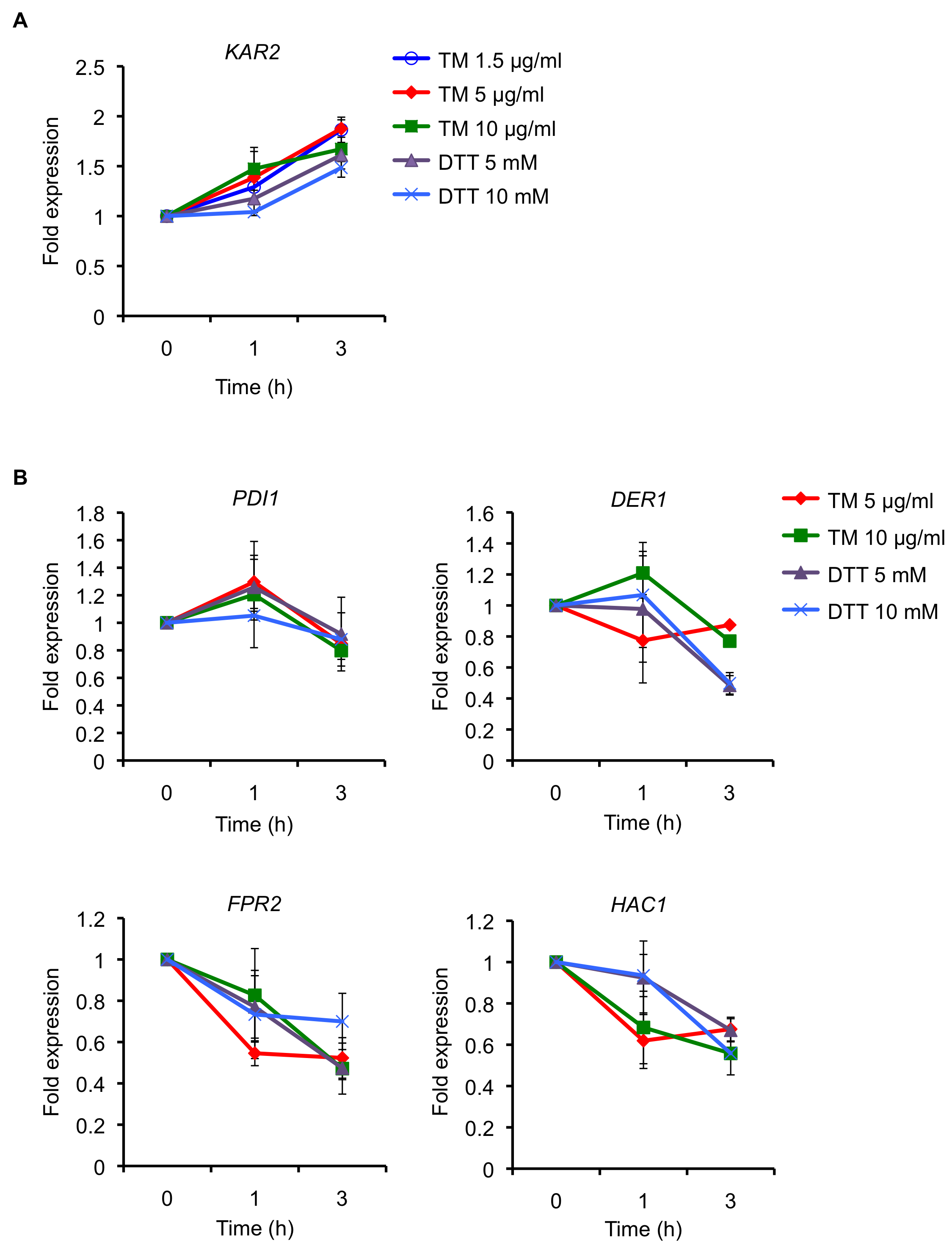

Supplement: Figure S4 — qRT-PCR analysis to validate expression profiles of representative UPR target genes in the presence of tunicamycin (TM) and dithiothreitol (DTT). C. glabrata wild-type (CBS138) cells were treated with TM or DTT at the indicated concentrations for 1 and 3 h. Expression levels of KAR2 (A) and other known UPR targets (B) were examined by qRT-PCR as described in Materials and Methods. The means and standard deviations for three independent experiments are shown. (TIF) [file ppat.1003160.s004.tif]

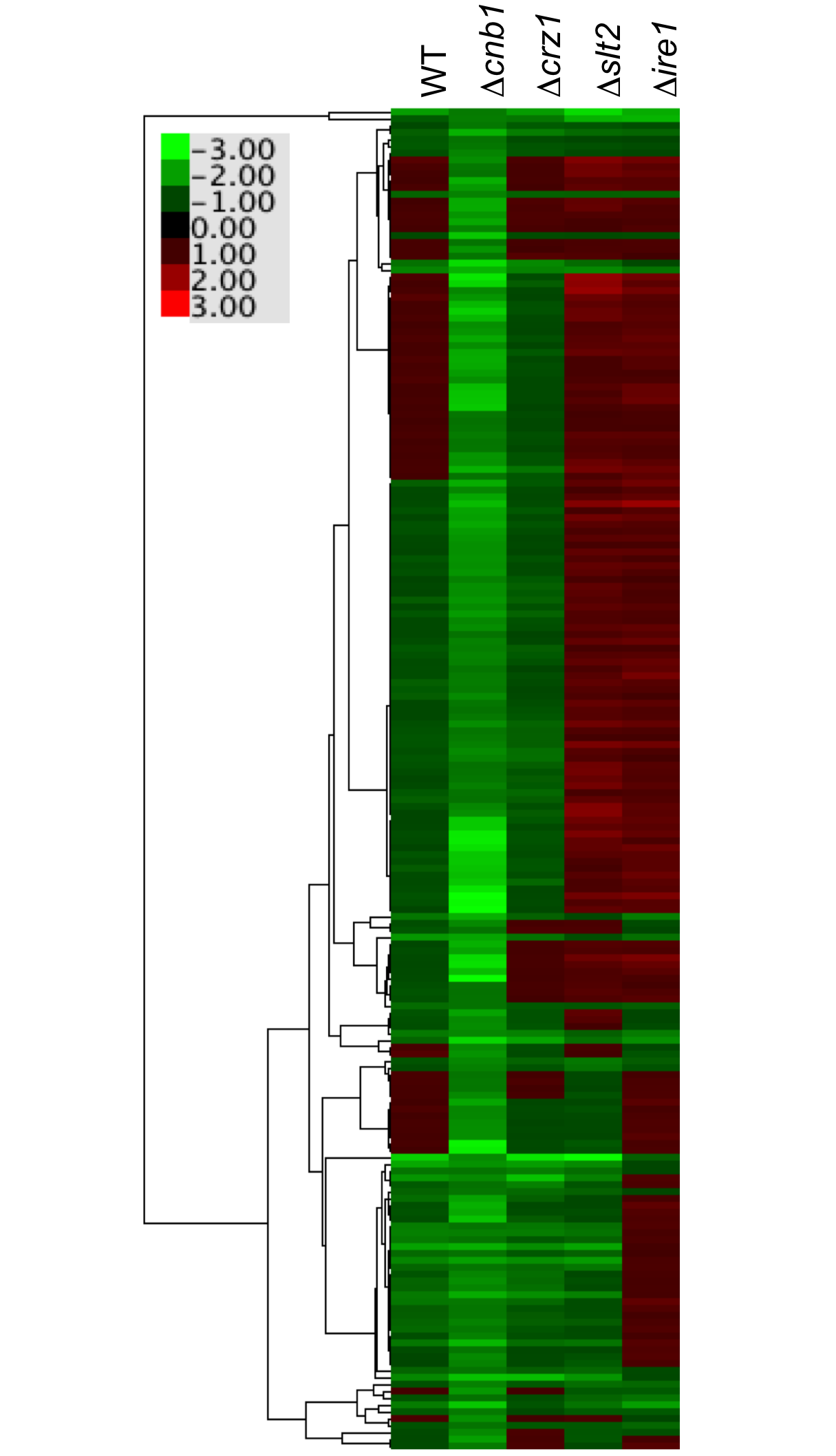

Supplement: Figure S5 — Genome-wide gene expression profiles in response to tunicamycin (TM) exposure for 0.5 h. Hierarchical clustering of genes whose expression levels were changed more than 1.5-fold after treatment with 1.5 µg/ml TM for 0.5 h. Genes were clustered with average linkage. C. glabrata strains: WT, 2001T; Δcnb1, TG161; Δcrz1, TG171; Δslt2, TG151; and Δire1, TG121. (TIF) [file ppat.1003160.s005.tif]

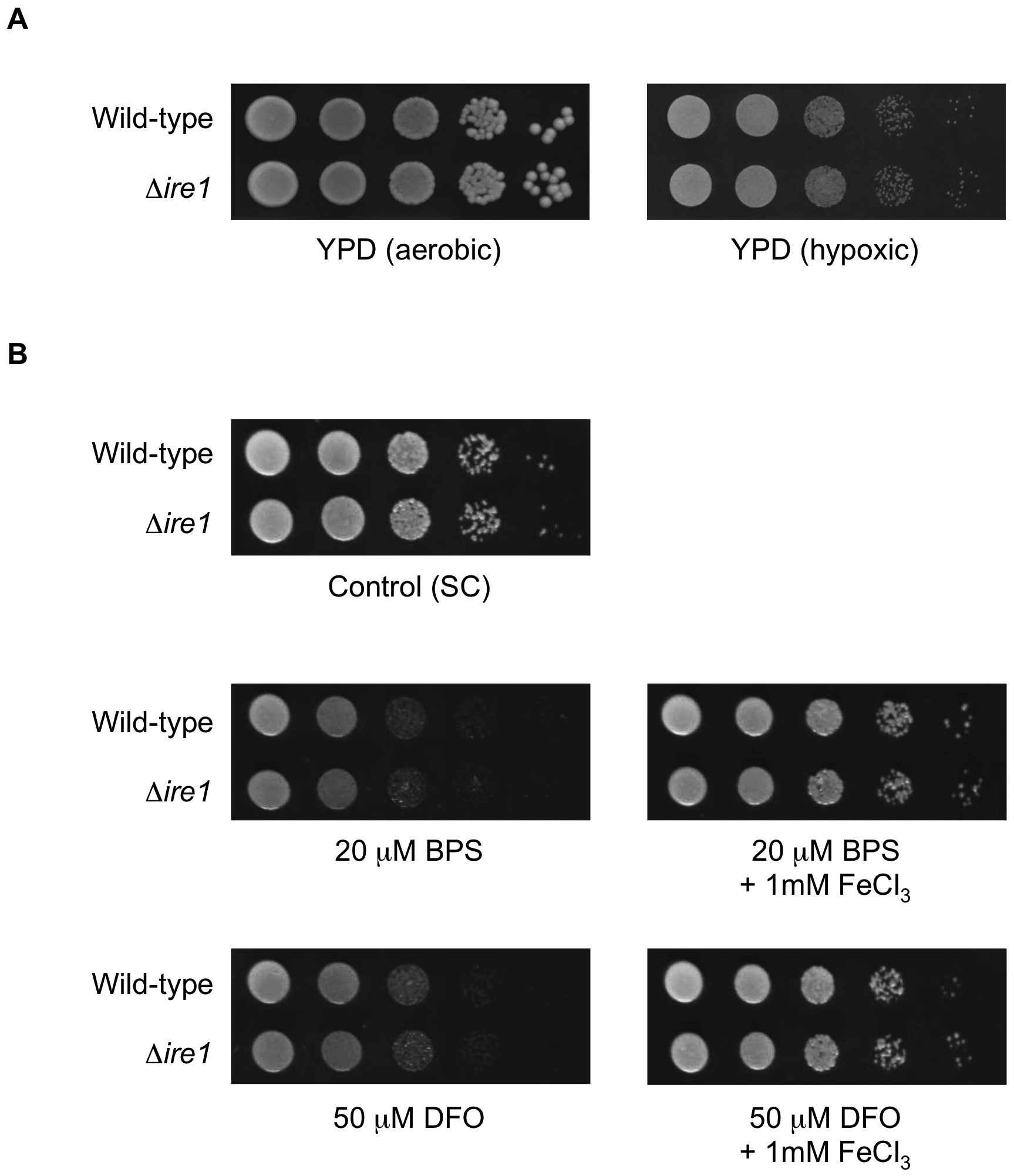

Supplement: Figure S6 — Ire1 is dispensable for growth under conditions of hypoxia and iron depletion in C. glabrata. (A) Logarithmic-phase cells of each C. glabrata strain were adjusted to 2×10^7^ cells/ml, and then 5 µl of serial 10-fold dilutions were spotted onto YPD plates. One plate was incubated under normal (aerobic) condition for 44 h, and the other plate was incubated under conditions of low oxygen tension (hypoxic) for 68 h. (B) Serial dilutions of C. glabrata cells were prepared as described above and spotted onto synthetic complete (SC) plates containing either 20 µM bathophenantroline disulphonate (BPS) or 50 µM desferrioxamine (DFO) in the presence and absence of 1 mM ferric chloride (FeCl3). Plates were incubated at 30°C for 24 h. (TIF) [file ppat.1003160.s006.tif]
