# Supplementary material for: Dissection of Ire1 Functions Reveals Stress Response Mechanisms Uniquely Evolved in Candida glabrata
Source: PLoS Pathog. 2013 Jan 31;9(1):e1003160. doi: 10.1371/journal.ppat.1003160 (PMC3561209; doi:10.1371/journal.ppat.1003160)
Supplement: Table S1 — Putative C. glabrata bZIP transcription factors identified by a BLASTp search using the bZIP domain sequences of S. cerevisiae Hac1, H. sapiens Xbp1, and C. neoformans Hxl1 as queries. (DOC) [file ppat.1003160.s007.doc]

**Table S1.** Putative *C. glabrata* bZIP transcription factors identified by a BLASTp search using the bZIP domain sequences of *S. cerevisiae* Hac1, *H. sapiens* Xbp1, and *C. neoformans* Hxl1 as queries.

|  |  | E-value | | |
| --- | --- | --- | --- | --- |
| *C. glabrata* Genorevules ID | *S. cerevisiae* ortholog | ScHac1 bZIP | HsXbp1 bZIP | CnHxl1 bZIP |
| CAGL0K12540g | *HAC1* (YFL031w) | 7e-08 | 0.005 | 0.005 |
| CAGL0H04631g | *YAP1* (YML007w) | 0.003 | 1e-07 | * |
| CAGL0J06182g | *SKO1* (YNL167c) | 0.005 | * | * |
| CAGL0K02585g | *YAP3* (YHL009c) | * | 6e-06 | * |
| CAGL0M10087g | *YAP3* (YHL009c) | * | 1e-04 | * |
| CAGL0L02475g | *GCN4* (YEL009c) | * | 2e-04 | * |
| CAGL0F03069g | *CAD1* (YDR423c) | * | 7e-04 | * |
| CAGL0F01265g | *YAP7* (YOL028c) | * | 0.002 | * |
| CAGL0M08800g | *YAP6* (YDR259c) | * | 0.008 | * |

ScHac1 bZIP, the bZIP domain of *Saccharomyces cerevisiae* Hac1 (37-95aa); HsXbp1 bZIP, the bZIP domain of *Homo sapiens* Xbp1 (48-132aa); and CnHxl1 bZIP, the bZIP domain of *Cryptococcus neoformans* Hxl1 (60-125aa). The asterisk (*) indicates e-value of > 0.01.
