# Supplementary material for: Dissection of Ire1 Functions Reveals Stress Response Mechanisms Uniquely Evolved in Candida glabrata
Source: PLoS Pathog. 2013 Jan 31;9(1):e1003160. doi: 10.1371/journal.ppat.1003160 (PMC3561209; doi:10.1371/journal.ppat.1003160)
Supplement: Table S6 — Strains used in this study. (DOC) [file ppat.1003160.s012.doc]

**Table S6.** Strains used in this study.

| **Strain** | **Genotype or description** | **Reference or source** |
| --- | --- | --- |
| ***C. glabrata*** |  |  |
| BG2 | Wild-type | [1] |
| ATCC 90030 | Wild-type | ATCC |
| CBS138 | Wild-type (ATCC 2001) | [2] |
| 2001T | Δ*trp1* | [3] |
| 2001HT | Δ*his3*, Δ*trp1* | [3] |
| 2001TU | Δ*trp1*, Δ*ura3* | [3] |
| 2001HTU | Δ*his3*, Δ*trp1*, Δ*ura3* | [3] |
| TG11 | 2001T containing pCgACT-P | [4] |
| TG13 | 2001T containing pCgACT-PScHAC(i) | This study |
| TG14 | 2001TU containing pEM14-GAS2 | This study |
| TG121 | Δ*ire1*::*HIS3*, Δ*trp1* (made from 2001HT) | This study |
| TG122 | TG121 containing pCgACT-P | This study |
| TG123 | TG121 containing pCgACT-PIRE | This study |
| TG124 | TG121 containing pCgACT-PIRE-KD | This study |
| TG125 | TG121 containing pCgACT-PIRE-ND | This study |
| TG126 | TG121 containing pCgACT-PScHAC(i) | This study |
| TG127 | Δ*ire1*::*HIS3*, Δ*trp1*, Δ*ura3* (made from 2001HTU) | This study |
| TG128 | TG127 containing pEM14-GAS2 | This study |
| TG141 | Δ*hac1*::*TRP1*, Δ*his3* (made from 2001HT) | This study |
| TG151 | Δ*slt2*::*HIS3*, Δ*trp1* (made from 2001HT) | [5] |
| TG152 | TG151 containing pCgACT-P | [5] |
| TG153 | TG151 containing pCgACT-PS2 | [5] |
| TG161 | Δ*cnb1*::*HIS3*, Δ*trp1* (made from 2001HT) | [4] |
| TG162 | TG161 containing pCgACT-P | [4] |
| TG163 | TG161 containing pCgACT-PNB | [4] |
| TG171 | Δ*crz1*::*HIS3*, Δ*trp1* (made from 2001HT) | [4] |
| TG172 | TG171 containing pCgACT-P | [4] |
| TG173 | TG171 containing pCgACT-PRZ | [4] |
| TG1412 | Δ*hac1*::*TRP1*, Δ*ire1*::*HIS3* | This study |
| TG1612 | Δ*cnb1*::*HIS3*, Δ*ire1*::*TRP1* | This study |
| TG1712 | Δ*crz1*::*HIS3*, Δ*ire1*::*TRP1* | This study |
| TG1512 | Δ*slt2*::*HIS3*, Δ*ire1*::*TRP1* | This study |
| ***S. cerevisiae*** |  |  |
| W303 | ATCC 200060 | ATCC |
| S288C | ATCC 204508 | ATCC |
| BY4742 | MATα *his3*Δ*1*, *leu2*Δ*0*, *lys2*Δ*0*, *ura3*Δ*0* | Open Biosystems |
| BY42-1 | BY4742 containing pRS415-ADH |  |
| BY42-2 | BY4742 containing pRS426-ADH |  |
| BY4742Δire1 | As BY4742, Δ*ire1*::*kanMX4* | Open Biosystems |
| BY42I-1 | BY4742Δire1 containing pRS415-ADH | This study |
| BY42I-2 | BY4742Δire1 containing pRS415-ADH-CgIRE1 | This study |
| BY42I-3 | BY4742Δire1 containing pRS426-ADH | This study |
| BY42I-4 | BY4742Δire1 containing pRS426-ADH-CgHAC1 | This study |
| BY42I-5 | BY4742Δire1 containing pRS426-ADH-ScHAC(i) | This study |
| BY4742Δhac1 | As BY4742, Δ*hac1*::*kanMX4* | Open Biosystems |
| BY42H-1 | BY4742Δhac1 containing pRS426-ADH | This study |
| BY42H-2 | BY4742Δhac1 containing pRS426-ADH-CgHAC1 | This study |
| BY42H-3 | BY4742Δhac1 containing pRS426-ADH-ScHAC(i) | This study |
| **Others** |  |  |
| *C. neoformans* H99 | Wild-type (serotype A MATα) | [6] |
| *C. albicans* SC5314 | Wild-type | [7] |
| *C. parapsilosis* ATCC 90018 | Wild-type | ATCC |
| *C. tropicalis* ATCC 750 | Wild-type | ATCC |
| *C. krusei* ATCC 6258 | Wild-type | ATCC |
| *C. guilliermondii* ATCC 6260 | Wild-type | ATCC |

ATCC, American Type Culture Collection, Manassas, VA.

1. Fidel PL, Jr., Cutright JL, Tait L, Sobel JD (1996) A murine model of Candida glabrata vaginitis. J Infect Dis 173: 425-431.

2. Dujon B, Sherman D, Fischer G, Durrens P, Casaregola S, et al. (2004) Genome evolution in yeasts. Nature 430: 35-44.

3. Kitada K, Yamaguchi E, Arisawa M (1995) Cloning of the Candida glabrata TRP1 and HIS3 genes, and construction of their disruptant strains by sequential integrative transformation. Gene 165: 203-206.

4. Miyazaki T, Yamauchi S, Inamine T, Nagayoshi Y, Saijo T, et al. (2010) Roles of calcineurin and Crz1 in antifungal susceptibility and virulence of Candida glabrata. Antimicrob Agents Chemother 54: 1639-1643.

5. Miyazaki T, Inamine T, Yamauchi S, Nagayoshi Y, Saijo T, et al. (2010) Role of the Slt2 mitogen-activated protein kinase pathway in cell wall integrity and virulence in Candida glabrata. FEMS Yeast Res 10: 343-352.

6. Perfect JR, Ketabchi N, Cox GM, Ingram CW, Beiser CL (1993) Karyotyping of Cryptococcus neoformans as an epidemiological tool. J Clin Microbiol 31: 3305-3309.

7. Gillum AM, Tsay EY, Kirsch DR (1984) Isolation of the Candida albicans gene for orotidine-5'-phosphate decarboxylase by complementation of S. cerevisiae ura3 and E. coli pyrF mutations. Mol Gen Genet 198: 179-182.
