# Supplementary material for: Dissection of Ire1 Functions Reveals Stress Response Mechanisms Uniquely Evolved in Candida glabrata
Source: PLoS Pathog. 2013 Jan 31;9(1):e1003160. doi: 10.1371/journal.ppat.1003160 (PMC3561209; doi:10.1371/journal.ppat.1003160)
Supplement: Table S7 — Primers used in this study. (DOC) [file ppat.1003160.s013.doc]

**Table S7.** Primers used in this study.

| **Primer *^a^*** | **Target** | **Sequence (5’ – 3’) *^b^*** |
| --- | --- | --- |
| **For gene deletion** |  |  |
| CgIRE1 100-F | *CgIRE1* | *GGCTACAGAACAAGTGTCATAGATTGATAACCGGACTCAAAAATTTACTGAATAAAAGGTTTGAGGATAACTTTCAAGTGATATTAGTATCTTGGATTA***TAATACGACTCACTATAGGGC** |
| CgIRE1 100-R | *CgIRE1* | *CAAGGTTGACCGCTCAATCGTTAATTATATACATGCGATTTACCTCATTAAATGATTATAGCATACTTTTTCAAAAGAACTCCTCGAATAAATTCTCAT***CGCTCTAGAACTAGTGGATCC** |
| CgHAC1 100-F | *CgHAC1* | *CCGAGCAGACTCTACCACCCTATACTGCCGGATACATATATCAATAACTCTACCAAGCTTTGATCCCTCATTGGAAATCTATCATTGGATATATTCCAT***TAATACGACTCACTATAGGGC** |
| CgHAC1 100-R | *CgHAC1* | *CTAAGAACATACTGTACTGGGTAATATCTTCTGAAGATATCAAAATCTTATTCTTTCTTATAACAGTTCTCTTAATTATTGACCACAATATATGGAAAG***CGCTCTAGAACTAGTGGATCC** |
| **For gene cloning** |  |  |
| CgIRE1-F1-Bam | *CgIRE1* | CGGGATCCATGGGTAAATTTTGGTTTTGGCC |
| CgIRE1-R(+203)-Xh | *CgIRE1* 3’UTR | CCGCTCGAGGCACAACAACAACACCAAC |
| CgHAC1-F(-25) | *CgHAC1* 5’UTR | TACCGAACTTGAACACCG |
| CgHAC1-R(+105) | *CgHAC1* 3’UTR | GCCCGCTAAGAACATACTG |
| ScHAC1-F1-Bam | *ScHAC1* | CGGGATCCATGGAAATGACTGATTTTGAACTAACT |
| ScHAC1-R717-Bam | *ScHAC1* | CGGGATCCTCATGAAGTGATGAAGAAATCATTCAATTC |
| CgGAS2-F(-646)-Kpn | *CgGAS2* 5’UTR | AAGGTACCCACCACCAAACATTATGAGG |
| CgGAS2-R53-Kpn | *CgGAS2* | AAGGTACCCTAAATAAAGCACTGGCAGC |
| **For mutagenesis** |  |  |
| CgIRE1-F2947 | *CgIRE1* | CATCACTATAATGACTTGCCAGAACAC |
| CgIRE1-R2916 | *CgIRE1* | GACTACTTTCTCCTTTTGGTATTTGC |
| CgIRE1-mut-F2171 | *CgIRE1* | TAAATCCACAAAATATTTTGATATCAAAATC |
| CgIRE1-mut-R2170 | *CgIRE1* | TATTACGGTGAATTATATTTAATGAATGTAAG |
| **For RT-PCR** |  |  |
| ScHAC1-F1 | *ScHAC1* | ATGGAAATGACTGATTTTGAACTAACT |
| ScHAC1-R717 | *ScHAC1* | TCATGAAGTGATGAAGAAATCATTCAATTC |
| CgHAC1-F1 | *CgHAC1* | ATGTCAGAATTTTCAGAAATGATTC |
| CgHAC1-R(+432) | *CgHAC1* 3’UTR | GAACGCTGAAAGCATTGC |
| **For Northern blot probe** |  |  |
| ScHAC1-F77 | *ScHAC1* | TGCCTCCAAGGAAAAGAGCC |
| ScHAC1-R387 | *ScHAC1* | ATCAGACGACGAGTGCGAAC |
| ScACT1-F219 | *ScACT1* | CGGTATTGTCACCAACTGGGAC |
| ScACT1-R701 | *ScACT1* | GAAGATTGAGCAGCGGTTTGC |
| CgHAC1-F83 | *CgHAC1* | GGATGCCTCCAAGAAAGAGAGC |
| CgHAC1-R527 | *CgHAC1* | GGTCTGACTGGGGTTGATGTTAC |
| CgACT1-F709 | *CgACT1* | CAAACCGCTGCTCAATCTTCC |
| CgACT1-R1133 | *CgACT1* | ATGGATGGACCACTTTCGTCG |
| **For qRT-PCR** |  |  |
| ScKAR2-F1921 | *ScKAR2* | TCTTTGTCCAAGGTCGCTTATCC |
| ScKAR2-R2023 | *ScKAR2* | CACCATCGTCATCTTCATCTTCG |
| ScPDI1-F477 | *ScPDI1* | CACTCCAGTTATCGTCCAATCCG |
| ScPDI1-R598 | *ScPDI1* | AATCATCGTCTGCGTTTTCAGC |
| ScDER1-F422 | *ScDER1* | GATTCTTCGGTGCCATTGATG |
| ScDER1-R632 | *ScDER1* | GGTGTTTCAGTGTTGCGGAAC |
| ScFPR2-F219 | *ScFPR2* | TGAACTTGGCGTTGGCAGAG |
| ScFPR2-R362 | *ScFPR2* | TCAGCACTTGGAGGAATGACG |
| ScACT1-F919 | *ScACT1* | CCAGGTATTGCCGAAAGAATGC |
| ScACT1-R1061 | *ScACT1* | TGTTGGAAGGTAGTCAAAGAAGCC |
| CgKAR2-F945 | *CgKAR2* | CGACTCCTTCGTTGATGGTATCG |
| CgKAR2-R1086 | *CgKAR2* | GATGTCCTTCTTCTCCAAGCCG |
| CgPDI1-F493 | *CgPDI1* | GGTGTCAACGACTTCAACGCTAC |
| CgPDI1-R627 | *CgPDI1* | TTCTTCAGCAGAGGAACCTTCG |
| CgDER1-F448 | *CgDER1* | CCACTGCTTGTTCCGTTTTACTTG |
| CgDER1-R610 | *CgDER1* | AATCGTATGGCGTCTTCGTGAG |
| CgFPR2-F256 | *CgFPR2* | TGGGAACAAGGTATCACAGGC |
| CgFPR2-R379 | *CgFPR2* | CAAAATCCAGCACAGCATTAGG |
| CgHAC1-F382 | *CgHAC1* | TCTACCTTAGCAGCCGAATGG |
| CgHAC1-R527 | *CgHAC1* | GGTCTGACTGGGGTTGATGTTAC |
| I8591-F291 | CAGL0I08591g | AAATGTGGGAAGCGACTCAGTG |
| I8591-R417 | CAGL0I08591g | TTGTTGTTGGTAGTGTGGCTGC |
| K11946-F164 | CAGL0K11946g | TAACAGCCTGGTTTGCTCC |
| K11946-R289 | CAGL0K11946g | CGAGCACTGGTTCATAAGC |
| M4191-F1281 | CAGL0M04191g | CGGATTCCACATCAACGCTC |
| M4191-R1436 | CAGL0M04191g | TCGTAGTTTTCCAGGTCGTAGACG |
| F4829-F63 | CAGL0F04829g | AGAGTCTAATGTTATCTCCGAGGGC |
| F4829-R195 | CAGL0F04829g | TTGTTGGGGCTGGAAATCG |
| M12320-F1700 | CAGL0M12320g | CAGCAGTTGTCTCATCTGTTATGGC |
| M12320-R1849 | CAGL0M12320g | GGTCGTCCTTCATCTGTTGGTAGC |
| A4081-F292 | CAGL0A04081g | GAACCAAGCACCACCATTACTACC |
| A4081-R400 | CAGL0A04081g | TAGGGGCAGACTTAGCGGAAAC |
| L6776-F2155 | CAGL0L06776g | GCCAAGTCAAACACAACATCTTCC |
| L6776-R2294 | CAGL0L06776g | CCACAAGCGTTACACAGCGTTC |
| I7249-F137 | CAGL0I07249g | GGATACCCGTGATAGTTGCCAAG |
| I7249-R302 | CAGL0I07249g | TACTGGTCCCACTCGTTGAAGC |
| CgECM33-F139 | *CgECM33* | AGCGGGTGTGAGACCATTGTTG |
| CgECM33-R256 | *CgECM33* | CCAAGGAGGTGGCATTGTAGATTC |
| CgGAS2-F1583 | *CgGAS2* | CCAAAAGCAACAAGAAGAATGCCG |
| CgGAS2-R1694 | *CgGAS2* | GCAAGAACAAATCCAAGACCAGC |
| CgGAS4-F1388 | *CgGAS4* | CCTCAGCAAAGTCCTCAAGTGTATC |
| CgGAS4-R1523 | *CgGAS4* | CCAAAGCCTGGAGTAGAAATCATC |
| Cg18S-F1187 | 18S rRNA | TGACTCAACACGGGGAAACTCAC |
| Cg18S-R1299 | 18S rRNA | CACTCCACCAACTAAGAACGGC |

*^a^* “Cg” and “Sc” stand for *C. glabrata* and *S. cerevisiae*, respectively. “F” and “R” indicate forward and reverse primers, respectively.

*^b^* Sequences homologous to flanking regions of the target ORF are shown in italics. Sequences shown in boldface are present in pBSK-HIS and pBSK-TRP. Restriction sites are underlined.
