# Supplementary material for: Dissection of Ire1 Functions Reveals Stress Response Mechanisms Uniquely Evolved in Candida glabrata
Source: PLoS Pathog. 2013 Jan 31;9(1):e1003160. doi: 10.1371/journal.ppat.1003160 (PMC3561209; doi:10.1371/journal.ppat.1003160)
Supplement: Table S8 — Plasmids used in this study. (DOC) [file ppat.1003160.s014.doc]

**Table S8.** Plasmids used in this study.

| **Plasmid** | **Description** | **Reference or source** |
| --- | --- | --- |
| pBSK-HIS | pBluescript II SK+ (Stratagene, La Jolla, CA) containing *C. glabrata HIS3* at the XhoI site | [1] |
| pBSK-TRP | A 1-kb XhoI fragment containing *C. glabrata TRP1* was excised from pCgACT and inserted into the XhoI site of pBluescript II SK+ (Stratagene). | This study |
| pGRB2.2 | *C. glabrata* centromere-based plasmid containing *S. cerevisiae PGK1* promoter, polylinker, *C. glabrata* *HIS3* 3’ UTR, and *S. cerevisiae* *URA3* | [2] |
| pCgACT | *C. glabrata* centromere-based plasmid containing autonomously replicating sequence and *C. glabrata TRP1* | [3] |
| pCgACT-P | A 1-kb SacI-KpnI fragment containing the *S. cerevisiae PGK1* promoter, polylinker and *C. glabrata HIS3* 3’UTR was excised from pGRB2.2 and inserted into the SacI-KpnI site of pCgACT | [1] |
| pCgACT-PNB | A 0.6-kb PCR fragment containing *C. glabrata* *CNB1* was inserted into the SmaI site of pCgACT-P | [4] |
| pCgACT-PRZ | A 1.9-kb EcoRI PCR fragment containing *C. glabrata* *CRZ1* was inserted into the EcoRI site of pCgACT-P | [4] |
| pCgACT-PS2 | A 1.6-kb SalI PCR fragment containing *C. glabrata* *SLT2* was inserted into the SalI site of pCgACT-P | [1] |
| pCgACT-PIRE | A 3.3-kb BamHI-XhoI PCR fragment (primers CgIRE1-F1-Bam/CgIRE1-R(+203)-Xh) containing *C. glabrata* *IRE1* was inserted into the BamHI-SalI site of pCgACT-P | This study |
| pCgACT-PIRE-KD | pCgACT-P containing a *C. glabrata* *IRE1* variant, in which two residues, D723 and K725, within the Ire1 kinase domain were mutated to asparagines | This study |
| pCgACT-PIRE-ND | pCgACT-P containing a *C. glabrata* *IRE1* variant, in which 10 residues (D973-Y982) within the Ire1 nuclease domain were deleted | This study |
| pCgACT-PScHAC(i) | A 0.7-kb BamHI PCR fragment (primers ScHAC1-F1-Bam/ScHAC1-R717-Bam) containing the induced form of *S. cerevisiae* *HAC1* (*ScHAC1^i^*) was inserted into the BamHI site of pCgACT-P | This study |
| pRS415-ADH | pRS415 containing the *S. cerevisiae ADH1* promoter | [5] |
| pRS415-ADH-CgIRE1 | A 3.3-kb BamHI-XhoI PCR fragment (primers CgIRE1-F1-Bam/CgIRE1-R(+203)-Xh) containing *C. glabrata* *IRE1* was inserted into the BamHI-XhoI site of pRS415-ADH | This study |
| pCRBlunt-CgHAC | A 1.1-kb *C. glabrata* *HAC1* region (primers CgHAC1-F(-25) and CgHAC1-R(+105)) was cloned into pCR-Blunt II-TOPO vector (Invitrogen, Carlsbad, CA) | This study |
| pRS426-ADH | pRS426 containing the *S. cerevisiae ADH1* promoter (in the SacI-XbaI site) | [5] |
| pRS426-ADH-CgHAC1 | A 1.1-kb EcoRI fragment containing *C. glabrata* *HAC1* was excised from pCRBlunt-CgHAC and inserted into the EcoRI site of pRS426-ADH | This study |
| pRS426-ADH-ScHAC1(i) | A 0.7-kb BamHI PCR fragment (primers ScHAC1-F1-Bam/ScHAC1-R717-Bam) containing the induced form of *S. cerevisiae* *HAC1* (*ScHAC1^i^*) was inserted into the BamHI site of pRS426-ADH | This study |
| pEM14 | *C. glabrata* centromere-based plasmid containing multiple cloning region, *lacZ*, and *S. cerevisiae* *URA3* | [6] |
| pEM14-GAS2 | A 0.7-kb KpnI fragment containing the 5’UTR of *C. glabrata* *GAS2* was inserted into the KpnI site of pEM14 | This study |

1. Miyazaki T, Inamine T, Yamauchi S, Nagayoshi Y, Saijo T, et al. (2010) Role of the Slt2 mitogen-activated protein kinase pathway in cell wall integrity and virulence in Candida glabrata. FEMS Yeast Res 10: 343-352.

2. Frieman MB, McCaffery JM, Cormack BP (2002) Modular domain structure in the Candida glabrata adhesin Epa1p, a beta1,6 glucan-cross-linked cell wall protein. Mol Microbiol 46: 479-492.

3. Kitada K, Yamaguchi E, Arisawa M (1996) Isolation of a Candida glabrata centromere and its use in construction of plasmid vectors. Gene 175: 105-108.

4. Miyazaki T, Yamauchi S, Inamine T, Nagayoshi Y, Saijo T, et al. (2010) Roles of calcineurin and Crz1 in antifungal susceptibility and virulence of Candida glabrata. Antimicrob Agents Chemother 54: 1639-1643.

5. Mumberg D, Muller R, Funk M (1995) Yeast vectors for the controlled expression of heterologous proteins in different genetic backgrounds. Gene 156: 119-122.

6. El Barkani A, Haynes K, Mosch H, Frosch M, Muhlschlegel FA (2000) Candida glabrata shuttle vectors suitable for translational fusions to lacZ and use of beta-galactosidase as a reporter of gene expression. Gene 246: 151-155.
